# Pathways to cultural adaptation: the coevolution of cumulative culture and social networks

**DOI:** 10.1017/ehs.2023.21

**Published:** 2023-08-25

**Authors:** Marco Smolla, Erol Akçay

**Affiliations:** 1 Department of Biology, University of Pennsylvania, Philadelphia, PA 19104, USA; 2 Department of Human Behaviour, Ecology and Culture, Max Planck Institute for Evolutionary Anthropology, Leipzig, Germany

**Keywords:** cumulative cultural evolution, social networks, social learning, heterogeneous environments, agent-based model

## Abstract

Humans have adapted to an immense array of environments by accumulating culturally transmitted knowledge and skills. Adaptive culture can accumulate either via more distinct cultural traits or via improvements of existing cultural traits. The kind of culture that accumulates depends on, and coevolves with, the social structure of societies. Here, we show that the coevolution of learning networks and cumulative culture results in two distinct pathways to cultural adaptation: highly connected populations with high proficiency but low trait diversity vs. sparsely connected populations with low proficiency but higher trait diversity. Importantly, we show there is a conflict between group-level payoffs, which are maximised in highly connected groups that attain high proficiency, and individual level selection, which favours disconnection. This conflict emerges from the interaction of social learning with population structure and causes populations to cycle between the two cultural and network states. The same conflict creates a paradox where increasing innovation rate lowers group payoffs. Finally, we explore how populations navigate these two pathways in environments where payoffs differ among traits and can change over time, showing that high proficiency is favoured when payoffs are stable and vary strongly between traits, while frequent changes in trait payoffs favour more trait diversity. Our results illustrate the complex interplay between networks, learning and the environment, and so inform our understanding of human social evolution.

## Graphical abstract

**Figure:**
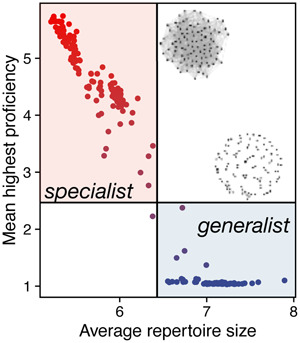

**Social media summary:** Letting culture and social networks co-evolve, we find two paths to cultural adaptation and a novel social dilemma.

## 1. Introduction

Our species’ ecological success is in large part based on our capacity to accumulate cultural knowledge (Boyd et al., 2011; Henrich & McElreath, 2003; Hill et al., 2009). We have accumulated vast amounts of cultural traits (e.g. knowledge, technologies, beliefs and ideas) that are too numerous or complex to be invented by a single individual (Boyd & Richerson, 1995; Henrich & McElreath, 2003; Mesoudi & Thornton, 2018). Instead, cumulative cultural adaptation proceeds by individual innovations that either add a new skill, tool or knowledge, or add to the complexity and efficacy of an existing one. These individual innovations, if they spread to enough individuals through social transmission, can be maintained in the population in the long term so that they do not have to be reinvented and can be built upon. In fact, our species’ unique cultural niche seems to be to build on various forms of social learning that allow us to specialise in resources that require high skill, and do so in a broad spectrum of environments (Hill et al., 2009; Kaplan et al., 2000; Roberts & Stewart, 2018). This capacity – to accumulate locally adaptive traits – enabled us to settle all over the globe from arid deserts to frigid polar regions, and from the rich equator to the relatively unproductive high latitudes (Boyd et al., 2011; Elton, 2008), and collectively adapt to changing environments and develop solutions to new problems (Galesic et al., 2023).

Whether and how much adaptive culture accumulates is a function of social and demographic parameters, such as group size (Derex et al., 2013; Henrich, 2004b), connectivity (Cantor et al., 2021; Derex et al., 2018), life span (Acerbi et al., 2012), acquisition costs (Mesoudi, 2011) and length of the learning period (Lehmann et al., 2010). The converse is also true: the quantity and quality of cumulative culture and the selection on cultural traits will determine selection on individual-level and group-level traits such as learning schedules (Lehmann et al., 2013) or network structure (Smolla & Akçay, 2019). Thus, understanding adaptation through cumulative culture needs to consider the reciprocal feedbacks between the dynamics of innovation and transmission of cultural traits and the individual and group-level mechanisms through which these dynamics occur.

An important feature of cumulative culture is that there are often many cultural traits that might be profitable, and each cultural trait can be built upon and improved by successive innovations. This gives individuals and societies multiple routes to increase their payoffs: (1) innovating and/or learning more profitable traits (i.e. having a large repertoire) or (2) innovating and/or learning improvements on traits that they already have (i.e. having high proficiency in fewer traits). Our previous work showed that when cultural selection favours large repertoires, groups evolve sparsely connected networks and large trait diversity, whereas selection for high proficiency results in densely connected groups that coordinate on a few traits, allowing successive innovations to be maintained in the group (Smolla & Akçay, 2019).

In the real world, groups and individuals can benefit from either accumulating more traits or higher proficiency – or both. Such open-ended cultural selection creates an inherent trade-off between learning new traits vs. improving proficiency on existing ones. As cultural traits and proficiency accumulate in the population, individuals will be constrained by limits of social learning, especially when social learning requires multiple exposures to the trait to be learned. The consequences of this inherent trade-off has not yet been explored. Most studies of cumulative culture rely on either accumulation of a single dimension of cultural complexity (e.g. Henrich, 2004a), with only a few studies (Kempe et al., 2014; Kolodny et al., 2015; Mesoudi, 2011) that consider the accumulation and improvement of different cultural traits. In most of these studies, however, there is no trade-off between learning new traits or maintaining and improving existing ones (Mesoudi, 2011, is a notable exception). Moreover, most models of cumulative culture regard social learning as a simple contagion process, assuming spread proportional to their local prevalence. This corresponds to an effective assumption that social learning happens or not instantaneously upon exposure. However, traits that make up adaptive cumulative culture, such as foraging tactics or making tools, are likely to require repeated exposures to be learned. This makes spread of cultural traits a non-linear function of their local prevalence. Our own previous work (Smolla & Akçay, 2019) incorporated this non-linearity and a trade-off at the individual level but sidestepped it at the population level by exogenously imposing selection for either only broad repertoire or only high proficiency. As a result, how individuals and societies navigate the competing pathways to cultural adaptation, and how these pathways coevolve with social structure, remain largely unknown. As we show here, this coevolution is subject to an unexpected emergent conflict between individual and group level cultural adaptation.

Another fundamental feature of cumulative cultural evolution is that it almost always happens in heterogeneous environments where the payoffs from different traits will be variable and fluctuate over time. Thus, cultural traits that are profitable in a given time and place might not be profitable in another (Boyd & Richerson, 1995; Richerson & Boyd, 2005). Such fluctuations can alter the trade-off between broader repertoires and higher proficiency: environmental change is expected to favour broader, more generalist cumulative culture as a way of bet-hedging (Deffner & Kandler, 2019), which in turn might be expected to favour individual innovation and cultural diversity. However, how cumulative culture coevolves with social network structure in heterogeneous and fluctuating environments remains unknown.

To address these gaps, we model open-ended cultural selection where individuals can increase their payoffs either by learning new cultural traits or increasing their proficiency in traits they already know. We first ask what kinds of social structure are produced by such open-ended cultural selection and find that, counter-intuitively, societies tend to cycle between densely connected states with high-proficiency culture and sparsely connected states with broad-repertoire culture. Interestingly, this cycling happens despite the fact that the specialist state has a much higher overall payoff. We show that cycling is driven by a conflict between group-level cultural adaptation and individual selection: average group level payoff is highest in highly connected, high proficiency cultures, but individual level selection favours sparser connections that eventually break apart the high-proficiency culture. Likewise, we show that increasing individual innovation rate inhibits high-proficiency culture through the same mechanism. We then ask what kind of social network structure and cumulative culture coevolve in heterogeneous and fluctuating environments where cultural traits have variable payoffs that change over time. We find that higher rates of environmental change and lower variation in payoffs between traits favour generalist societies with sparse network connections, while slower environmental change and higher trait variance favour specialist and well-connected societies. These results illustrate the complex interplay between group structure, social learning and environmental variation in cumulative cultural adaptation.

## 2. Methods

We consider a population of *N* asexually reproducing individuals, with overlapping generations in a world with *T* learnable traits or skills. These skills relate to subsistence, social norms or other aspects relevant to an individual's survival. As we are interested in the effect of environmental heterogeneity on social structure and cultural evolution, we assume that skills differ in their utility. A skill's utility, *u*, translates into a payoff that an individual receives if the skill is part of its repertoire. The values of *u* are randomly drawn for each trait, *t*, from lognormal distributions, lognormal(*μ*, *σ*^2^) in the interval [0, 10]. If not stated otherwise we use *μ* = 0 and *σ*^2^ ∈ {0.2, 0.4, …, 1}, and so mainly varying payoff variance (see Supplementary Material, Figure S1). We simulate environmental change as a change in skill utilities across time. In our model, time is divided into rounds. In each round each skill has a small probability of receiving a new randomly drawn utility from the same distribution the simulation was started with. We express the environmental change probability as the number of updated utilities per generation (i.e. every *N* rounds) *τ* ∈{10^−3^, 10^−2^, …, 10^1^}. In fast changing environments (*τ* = 10^1^), 10 utilities are updated every generation. At the other extreme (*τ* = 10^−3^) it takes on average 1000 generations for a single utility to change. In the latter case the environment is essentially fixed. In the former case, payoffs change, for example, because certain resources are ephemeral and a trait that allows use of them is only useful for a short amount of time. While these interpretations assume a sessile population that experiences a change in their environment, an equally plausible interpretation is that of a population on the move that encounters different environments.

Population turnover follows a Moran death–birth process (Moran, 1958), that is, in each simulation round: (1) one individual is removed from the population (selected relative to the inverse of their payoff, i.e. mortality selection); (2) one of the remaining individuals is randomly selected to be a ‘parent’ and a copy (subject to mutation of the connection traits) with an empty repertoire is added to the social network; and (3) the new individual acquires cultural traits and proficiency through innovation (individual learning) or social learning from its network neighbours. We refer to *N* death–birth events as one generation, and so the rate of replacement scales with population size *N*. In the following sections, we discuss these steps in more detail.

### 2.1 Population structure

We use complex dynamic networks structured by social inheritance (Ilany & Akcay, 2016) to simulate population structure and turnover. These networks capture important aspects of real-world networks (Ilany & Akcay, 2016) and allow the dynamic formation of local and global clustering in response to different selective regimes. The social inheritance model has three linking parameters, which represent the probabilities that a new individual (1) forms a connection with its parent, *p*_b_ (here, we assume *p*_b_ = 1), (2) with the neighbours of the parent, *p*_n_ and (3) with other individuals that are not connected to the parent, *p*_r_. A new individual that is added to the population inherits *p*_n_ and *p*_r_ vertically from its parent (culturally or genetically see, e.g. Brent et al., 2013). Mutation occurs with probability *m* = 0.05, whereby mutated values are drawn from a normal distribution centred around the parent's value with standard deviations 0.05 and 0.005 for *p*_n_ and *p*_r_ respectively. Note that the SD for *p*_r_ is smaller, because the number of potential socially inherited links is generally much smaller than the number of potential random links, and so values of *p*_r_ are smaller by an order of magnitude. The new individual is connected to its parent and to other individuals based on *p*_n_ and *p*_r_.

### 2.2 Learning

Next, the new individual enters the learning phase, allowing her to either acquire new skills or improve skill proficiency. The learning phase consists of 100 social learning and 100 innovation attempts. At birth, an individual's proficiency *l* is zero for all *T* skills. Skill proficiency increases through successful learning. When a new skill is acquired through innovation or copying, the proficiency of skill *t* increases by one unit, i.e. l_t_* = *l_t_* + 1. An individual's repertoire size *R_i_* is the number of non-zero skill proficiencies *l*. To become better at performing a skill, repeated engagement with it is required, as learning takes time (Karni et al., 1998; Karni & Sagi, 1993; Lew-Levy et al., 2017; Morelli et al., 2003). Therefore, proficiency increases with each successful individual or social learning attempt of the same skill. An individual's highest proficiency *L_i_* is equal to the largest *l_t_* in its repertoire. Because the number of learning turns is limited and attention to one skill limits attention to other skills, there is a trade-off between becoming good at a skill and learning many skills. Hence, there is a negative relationship between skill proficiency and repertoire size.

During an *individual learning* episode the individual first picks one skill from all possible skills *T* at random, and then attempts to acquire proficiency for this skill. Learning success is moderated by an innovation success probability, *α*, and so the probability of acquiring *t* through innovation is1
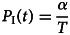


During a *social learning* episode the individual first picks a skill from those performed in its vicinity (i.e. present in the repertoire of her neighbours). Given that each individual is assumed to be equally likely to perform any of their traits, the probability that individual *i* observes trait *t* in her neighbourhood is *p_i_*_,*t*_ = *n_i_*_,*t*_*R_n_i__^−^*^1^, where *n_i_*_,*t*_ is the number of *i*'s neighbours with skill *t*, and *R_n_i__* is the sum of repertoire sizes of *i*'s neighbours. Subsequently, the newborn attempts to acquire proficiency for this skill. Learning success is moderated by a social learning success probability, *β*. Crucially, we assume that social learning is more effective when an individual receives more exposure to a skill, i.e. if *p_i_*_,*t*_ is larger, and so the probability of acquiring *t* through copying is:2
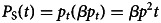


Equation (2) can be thought of as complex contagion in a given trait (Centola, 2010), where transmission depends on the probability of observing a skill twice, regardless of the individual being observed. As pointed out previously (Smolla & Akçay, 2019), this relates to Simpson's (1949) index, and so as skill diversity in *i*'s neighbourhood increases the exposure to each skill decreases, making it less likely to be observed sufficiently for successful social learning. Thus, equation (2) shows that acquiring a skill socially is more likely if social learning is easy (large *β*), *t* is common among neighbours (large *n_i_*_,*t*_), and/or if neighbours possess few skills (small *R_n_i__*). Additionally, we assume that an individual cannot surpass the proficiency of the observed individuals by social learning, and thus *P*_S_(*t*) = 0 where all neighbours have proficiency equal to or less than that of the individual *i* for skill *t*.

### 2.3 Payoff

After the learning phase, we calculate the total payoff *W*, which an individual receives from each skill in its repertoire. We calculate *W_i_* as the sum of the product of the skill proficiencies in *i*'s repertoire and their utility:3
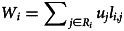


Because of the trade-off between learning many different skills or becoming very good in a few skills, equation (3) can be maximised in two different ways: either by acquiring some proficiency in many well-paying traits (hereafter *generalist*) or by becoming proficient in the most profitable skills (hereafter *specialist*). A homogeneous environment (where *u_j_* is the same for all *j*), learning a new trait or improving proficiency yields the same payoff, so our utility function does not inherently favour one or the other pathway.

We model here mortality selection owing to cultural traits, where the *W_i_* of an individual determines its probability of being selected to die in every time period. Note that this is different from Smolla and Akçay (2019), where selection is on fertility, i.e. the payoff owing to culturally acquired traits affected the probability of getting selected to reproduce. Implementing fertility or mortality selection has no effect on our main results in homogeneous environments (see Figure S16); all results discussed in the main text for mortality selection also carry over to fertility selection. However, in heterogeneous environments fertility selection can result in densely connected populations ‘getting stuck’ on a few traits, an ‘echo-chamber’ phenomenon as observed and discussed by Smolla and Akçay (2019). This effect makes densely connected populations unable to track changing environments.

The payoff *W_i_* in equation (3) is in principle unbounded, but in practice will be limited by the finite individual and social learning success of individuals. However, in heterogeneous environments with high trait variance, a few very valuable traits (very high *u_j_*) might end up having a disproportionate effect on survival in our simulations and drive artefactual results. To guard against such a nuisance outcome, we assume that fitness exhibits diminishing returns with utility. Specifically, we assume the probability of death is proportional to 1/*M*(*W_i_*), where *M*(*W_i_*) is a Michaelis–Menten function *M*(*W_i_*) = 1 + (*V*_max_*W_i_*)(*K* + *W_i_*)^−1^, with upper limit *V*_max_ = 50, a half rate constant *K* = 50 and a minimum payoff of 1. The high value of the half-rate constant means that, for most of the parameter range, this probability increases almost linearly with *W_i_* while guarding against unrealistic strong selection effects with extreme heterogeneity in trait payoffs.

A simulation turn ends with re-calculating each individual's payoff. A new simulation round starts with the removal of an individual, and selection of a random survivor.

### 2.4 Population size

The effect of population size on cultural evolution has been somewhat controversial recently (see e.g. Fay et al., 2019; Henrich, 2004b; Powell et al., 2009; Shennan, 2001). We consider how population size affects the coevolution of culture and network structure by simulating populations of different sizes, *N* ∈{25, 50, 75, 100, 200}.

### 2.5 Simulation parameters

If not stated otherwise, we run all simulations with *N* = 100 and *M* = 500, for 5000 generations (data being averaged over the last 200 generations to remove simulation artefacts from the initialisation of each simulation) and 200 repetitions, with mutation rate *m* = 0.05 (with SD = 0.05 for *p*_n_ and SD = 0.005 for *p*_r_), innovation success rate *α* = 0.02 and social learning success rate *β* = 0.5. Note that we chose *M* to be as large as needed for populations to never reach it but as small as possible for computational convenience. Complex networks are initialised with random values drawn from uniform distributions (*p*_n_, *U*(0, 1); *p*_r_, *U*(0, 0.1)). Individuals are initialised with empty repertoires. For robustness checks see the Supplementary Material Section S2.2.

### 2.6 Outcome variables

To compare differences in cultural knowledge between populations we record average repertoire size (
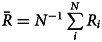
) and mean highest per individual trait proficiency (
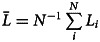
). To compare evolved networks, we record mean degree centrality and average weighted component size. Mean degree centrality, a measure for connectedness, is the average number of connections individuals have, where higher mean degree centrality signifies more connections between individuals, and vice versa. As the emerging networks can have unconnected components, we calculate the average weighted component size as a measure for the component size distribution. We define average weighted component size as the sum of the product of the component size, *c_s_*, and the relative component size, *c′_s_* over all component sizes, that is 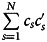. We calculate the relative component size 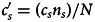, where *n_s_* is the number of components of size *s*, which is equivalent to 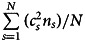. If not stated otherwise, all reported results are averages of the last 20% of generations.

### 2.7 Software

The simulations were run with Julia 1.8.5 (Bezanson et al., 2017), and analysed with R 4.2.2 (R Core Team, 2022).

## 3 Results

### 3.1 Homogeneous environments

#### 3.1.1 There are two distinct pathways to cultural adaptations

**Figure 1.**
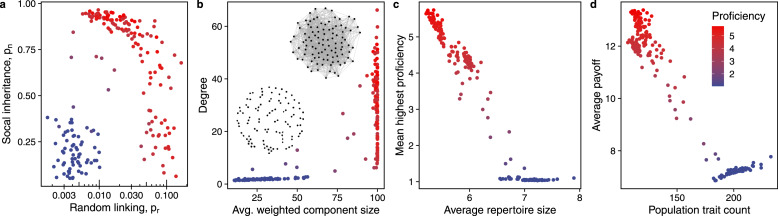
The two pathways to cultural adaptation. The four panels depict 200 populations from our simulations, with each panel showing different characteristics of the same set of populations. Each population is represented by a dot, coloured according to the mean proficiency of that population. When linking parameters evolve two distinct kinds of populations emerge: low vs. high mean linking traits (*p*_n_ and *p*_r_, panel a), low degree and small, unconnected components vs. high degree and a single connected component (panel b), large repertoires and low proficiency vs. smaller repertoires and high proficiency (panel c), and high trait diversity and low payoff vs. low trait diversity and high payoff (panel d). Simulations with *α* = 0.01, *β* = 1, *N* = 100, *M* = 500, running for 5000 generations, *τ* = 0 and *σ* = 0.

We find that two distinct types of populations emerge in homogeneous environments (Figure 1). The first type has low average connection probabilities (*p*_n_, *p*_r_) and features sparsely connected (low degree) networks with disjointed components (average component size less than *N*). The second type of population has high average connection probabilities and densely connected networks composed of a single connected component (average component size equals *N*; see representative examples as insets in Figure 1b). These populations display two distinct kinds of cumulative culture as characterised by the average repertoire size and average skill proficiency. Sparser networks only reach baseline proficiency on average, but have a slightly larger repertoire (Figure 1c). In contrast, denser networks achieve higher average proficiency with only a slightly reduced average repertoire. Interestingly, we find that proficiency is high for a wide range of degrees, so long as the network is connected. In contrast, repertoire sizes are large for a wide range component sizes, as long as average degree is low and there are at least a few unconnected components.

While average repertoire sizes at the individual level differ only modestly between the two kinds of populations, the cultural makeup at the population level differs markedly. Populations with sparser networks possess almost twice as many unique traits compared with those with denser networks but they also have much lower average payoff at the same time (Figure 1d). Further, the trait frequency spectrum differs. Populations with sparse networks have a much more even distribution with many traits at intermediate frequencies, whereas dense networks converge to a few traits known to almost every individual and a high number of traits at low frequencies (Figure S2). The convergence to a few traits by the whole population allows these networks to increase proficiency in these traits.

#### 3.1.2 Low-payoff state persists due to cycling

**Figure 2.**
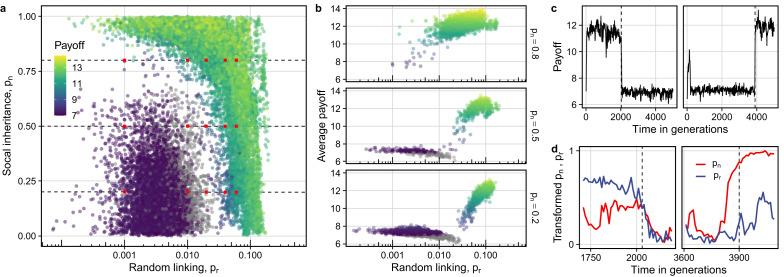
The fitness landscape for connection traits, *p*_n_ and *p*_r_. Panel a shows mean linking parameters and payoff for populations (one every 10 generations over the last 100 generations). The distribution of lighter and darker coloured points highlights the distinct population states separated by a fitness valley where the populations spend negligible time (for illustrative purpose payoffs *<* 7 shown in grey). Panels in b depict cross-sections of this fitness valley at particular values of *p*_n_ (at the horizontal dashed lines in panel a). The red dots depict the resident *p*_n_ and *p*_r_ values used for Figure 3. The results in c and d are two example simulations where the first shows a down-transition from the high payoff state and the second an up-transition from the low-payoff state. The changes in the linking parameters in (d) show how the down-transition begins with a drop in *p*_r_ followed by a drop in *p*_n_, whereas it is the opposite for the up-transition (for more details see the Supplementary Material, Figure S9). Simulations with *α* = 0.01, *β* = 1, *N* = 100, *M* = 500, running for 5000 generations, *t* = 0 and *s* = 0.

Figure 1d shows that groups with larger repertoires have on average much lower payoffs than those with high skill proficiency. Why do high-repertoire populations persist in the long term despite this payoff disadvantage? Analysing simulation trajectories, we find that populations exhibit cycles in the *p*_n_–*p*_r_ space (Figure S8) that repeatedly push them between the high-proficiency (and high payoff) and large-repertoire (and low payoff) states (videos of cycling behaviour available in the Supplementary Material). This cycling is a result of how different combinations of *p*_n_ and *p*_r_ relate to average payoff. Figure 2 shows the average linking parameters and associated average payoffs for 100 simulations over their last 100 generations. The density of the points in Figure 2 indicates where the populations spend their time in steady state. This plot highlights two important features: first, the average payoff stays high as long as either *p*_n_ or *p*_r_ or both are high. Second, there is a region separating the low- and high-linking probabilities that populations do not linger in. Plotting average payoffs across constant *p*_n_ transects in Figure 2b shows that this region corresponds to a ‘valley’ of low average payoff. Specifically, average payoff decreases with decreasing *p*_r_, but increases somewhat again at very low *p*_r_. Yet time trajectories from our simulations show that populations consistently drop into this payoff valley from the high-payoff state (i.e. from the right-hand side with low *p*_n_) and emerge on the other side, in the low-payoff state (on the left-hand side, see also Figure S9).

#### 3.1.3 Cycling results from the conflict between individual and group payoff

**Figure 3.**
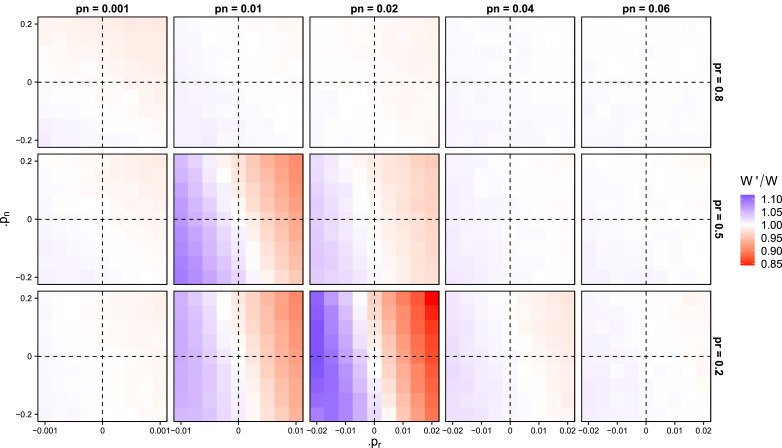
Local selection pressures on the linking traits. Each panel corresponds to results from 10 populations fixed for a particular value of *p*_n_ and *p*_r_ (given on the right-hand side and top of the grid, respectively; these correspond to the red dots in Figure 2). For each panel we initialise 10 populations by running our model with fixed connection traits and allowing the cumulative culture to come to a steady state. Then we introduce a single mutant that deviates from the resident linking parameters by Δ*p*_n_ and Δ*p*_r_, which are depicted on the *x*- and *y*-axes of each panel, respectively. Next, we calculate the relative payoff *W*′/*W* of the mutant relative to the mean payoff of the residents, as a result of the cultural traits the mutant learns and innovates. We repeat this 500 times for every combination of Δ*p*_n_ and Δ*p*_r_. Relative fitness is generally higher with lower *p*_n_ and *p*_r_, revealing individual level selection for disconnecting, despite the population level consequences for group level payoff depicted in Figure 2. Simulations with *α* = 0.01, *β* = 1, *N* = 100, *M* = 500, running for 5000 generations, *τ* = 0 and *σ* = 0.

To explain this puzzling observation, we computed the local selection pressures acting on the linking traits *p*_n_ and *p*_r_ in populations that are fixed for a particular value of the linking traits and have the associated steady state cumulative culture. Figure 3 depicts the relative payoff (dis-)advantage of a mutation that changes the linking traits for an array of resident *p*_n_ and *p*_r_ values. This gives the local selective landscape at the individual level across the *p*_n_–*p*_r_ space and reveals a striking contrast between individual selection and population average payoff. Even though average payoffs at the population level are highest when either *p*_n_ or *p*_r_ or both are high, selection almost always favours reduced linking at the individual level.

This discord between average group payoff and individual-level selection emerges from the interaction between emergent cumulative culture and social learning as we model it. Recall that high-payoff populations have dense networks and strong cultural convergence (that is, a small number of traits that are present in all repertoires and for which there is high proficiency). At the same time, because every individual also innovates random traits with a small probability, being connected to many others brings the individual in contact not only with the most common traits but also with those that are rare. However, because learning in our model requires repeated observations of a trait, the presence of a large number of rare traits reduces the overall success of social learning. This ‘distraction’ effect of rare traits applies most strongly to other rare traits (as common traits are observed at the same overall frequency no matter how many connections are made). Therefore, individuals with lower *p*_n_ and/or *p*_r_ (who on average connect to fewer others) will be more likely to learn more socially and have a higher payoff. This individual benefit drags the population towards the valley in the average fitness, and ultimately to the low-payoff state. Figure 3 shows that the strongest pull towards the low payoff population state occurs in the fitness valley described above. Populations can escape from this sparsely connected, low-payoff state either by drift, owing to the weak selection with low *p*_r_, or by trading random connections for socially inherited ones: at low *p*_n_ and *p*_r_ (lower left panels in Figure 3), individual selection can favour increased *p*_n_ as long as it is combined with decreased *p*_r_. This is because an increase in social inheritance links the individual to a clustered neighbourhood that is more likely to be similar in their trait repertoire. This creates local cultural convergence and reduces the number of unique rare traits an individual is exposed to. These clustered components can thus achieve higher payoffs and grow to take over the population, a kind of component-level selection that takes populations back to the connected, high-proficiency state.

Transitions between high and low payoff states happen more often in smaller populations than large populations (see Table S1) and larger populations spend more time in the high payoff state in the long run. However, even relatively large populations (*N* = 500) can spend 30% of time in the low-payoff state given our base parameters.

This cycling behaviour is a previously unrecognised example of a social dilemma where the interest of the individual is at odds with that of the group. The group benefits from being highly connected, as this allows for cultural convergence and subsequently an increased skill proficiency. While the individual benefits from this situation, they benefit even more by reducing their linking to the group. However, ultimately, this results in the social networks breaking apart and a loss of accumulated proficiency that disconnected networks cannot produce or sustain. This is a new kind of social dilemma between cultural adaptation at the group level and selection for the social structure that supports it at the individual level. Crucially, the assumption that social learning requires repeated exposures is necessary for these results, since this assumption creates a trade-off between overall success of social learning and the number of traits. If social learning can follow from single exposures, there is no such trade-off, and accordingly, populations evolve to be highly connected but without the convergence on a few traits and associated high proficiency (see Figures S10 and S18).

#### 3.1.4 Frequent innovation selects for sparser networks and lower payoff

**Figure 4.**
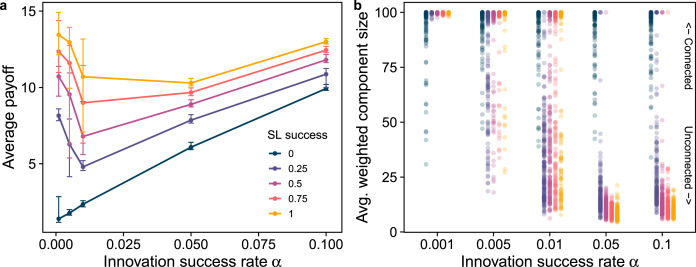
The rate of innovation and social learning affects payoffs (a) and social network structure (b). Panel (a) depicts average payoffs as a function of innovation success rate *α* for different social learning success rates (*β*). In the absence of social learning (*β* = 0), we find that increasing innovation always increases payoffs. However, with social learning (*β >*0), we find that average payoffs decrease with innovation rate initially before recovering at high innovation rates. Panel (b) depicts the average weighted component size, a measure of the connectivity of the network, with social learning success rate *β*, for different values of individual learning rates. It shows that as long as there is any social learning (*β >*0) higher individual innovation rate results in less connected networks. Simulations with *N* = 100, *M* = 500, running for 5000 generations. Error bars in a represent 90% confidence intervals.

Next, we explore how individual innovation and social learning rates influence the coevolution of network structure and cumulative culture. If there is no social learning (*β* = 0), increasing innovation rate *α* always increases average payoff (Figure 4a), independent of the environment. This is straightforward because without social learning, an individual's repertoire and proficiency depends solely on innovation, and more innovation means both larger repertoire size and higher proficiency. Social network structure, as expected, evolves neutrally in this case (Figure 4b).

In the presence of social learning, however, we again find an unexpected result: increasing individual innovation rate *α* decreases the average payoffs of the population (Figure 4a). This counter-intuitive pattern again happens because of the interplay between network structure and social learning: when innovation rates are low, individuals carry few or no rare idiosyncratic traits. Therefore, connecting to more individuals does not carry a cost in terms of reduced learning probabilities, which reduces the individual level selection to disconnect. Populations thus remain highly connected, converge on a few traits, and retain improvements in proficiency in these even if they are very rare. As individual innovation rates increase, however, each individual innovates more traits, which in a highly connected population reduces the probability of acquiring traits through social learning. Therefore, the individual level selection for reduced connections becomes stronger, and as a result, populations are more likely to spend time in the sparsely connected, low payoff state (Figure 4b). As innovation rates increase further, populations spend all their time in the sparsely connected state, and average payoff only recovers when innovation rates are so high that individual innovation starts compensating for the lack of socially learned traits. Note that increased individual innovation rate is always directly beneficial for the innovating individual (as it simply increases its proficiency or repertoire), but our results illustrate it can inhibit accumulation of culture because of its effect on network evolution, another instance of a conflict between individual selection and group-level cultural adaptation.

### 3.2 Heterogeneous environments

#### 3.2.1 Environmental turnover and trait variance affects the networks that evolve

**Figure 5.**
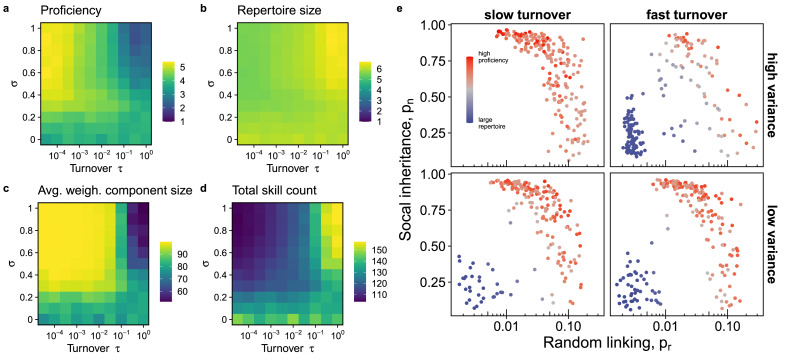
Environmental heterogeneity affects the viability of the generalist and specialist repertoire populations. When linking parameters evolve in different environments, we find stronger reliance on high proficiency (and there are fewer traits in the population overall) where turnover is low and utilities are highly skewed (large *σ*), whereas in environments with frequent turnover we find stronger reliance on larger repertoires. Simulations with *α* = 0.01 and *β* = 1, *N* = 100, *M* = 500, running for 5000 generations, averaged over 200 repetitions. False colour scale in (e) is based on the following data transformation of average repertoire size (*R*) and highest proficiency (*L*): *L*/max(*L*) - *R*/max(*R*). Subset of data in (e) with *t* ∈{10^−^^3^,1} and *σ* = {0.2,1}. See Figure S11 for additional results.

In heterogeneous environments, where payoffs vary across traits and time, we find that the variance in trait payoffs and the turnover rate of payoffs play a crucial role in determining the network structure and associated cumulative culture. Specifically, high proficiency culture evolves much more readily in stable environments with highly skewed utility distributions (Figure 5a), which coincides with occurrence of connected graphs (Figure 5c). In contrast, environments with fast environmental turnover but highly variable utilities favour the evolution of sparse networks with larger individual skill repertoires (Figure 5b,c) and a larger cultural repertoire (Figure 5d). Here, coordination on a few traits and accumulation of proficiency does not happen fast enough before payoffs change again. These differences disappear as utility distributions become increasingly uniform (small *σ*). That said, similar to the homogeneous environment case, we find both kinds of networks and their associated repertoire type (Figure 5e) in the majority of environments that we tested. Only where utilities are highly heterogeneous and environments are stable is the low payoff state absent.

#### 3.2.2 Larger populations accumulate more proficiency in stable environments

Finally, we vary population size and find that as populations become larger they are more likely to accumulate higher proficiency and be more connected (Figure S13). However, this feature is conditional on environmental stability. Where utilities change frequently, we do not find an effect of population size: larger populations stay disconnected and in the low proficiency, broad repertoire state. It is interesting to note that the cumulative nature of learning in our model allows individuals to reach higher proficiency in larger populations. However, this is not the case for repertoire size. In fact, individuals in unconnected graphs of large populations achieve similar if not smaller repertoire sizes compared to smaller populations (Figure S15).

## 4 Discussion

Our results uncover some fundamental tensions in the coevolution of cumulative culture and social network structure through which cultural traits are propagated. We demonstrate two distinct pathways to cultural adaptation are available to populations in a setting where both learning new traits and increasing proficiency in existing traits are possible. These two pathways (broad repertoire vs. high proficiency) correspond to distinct population structures and cumulative culture features. At the same time, we uncover two general conflicts that populations face when navigating these pathways. First, we show that although groups do best on the aggregate when they are well connected, coordinate on a few core cultural traits and build up proficiency, selection favours disconnection at the individual level. This creates a previously unrecognised type of conflict between individual and group-level payoff. Second, we show that increasing individual innovation rates results in less connected networks and a lower population level payoff. This is because all else being equal, higher individual innovation increases the trait diversity around an individual which again makes social learning less successful, and increases the selection against social connections. Since individual innovation is also individually beneficial (as it only results in individuals knowing more traits or having higher proficiency), this is another dimension of conflict between individual and group-level payoff. It is important to note that both of these results obtain despite there being no inherent cost to making or maintaining social connections.

Both of these conflicts between group- and individual-level benefits arise from the nature of social learning. Our model does not inherently privilege learning or innovating new traits over increased proficiency in an existing trait. However, individuals can only learn socially when they are repeatedly exposed to the same trait. Having more connections exposes an individual to more idiosyncratic traits that have been individually innovated, which reduces the probability of successful social learning overall. A similar negative effect of being exposed to too many options on social learning success has been reported in a paper-plane transmission chain experiment: Fay et al. (2019) found that being exposed to only one model (vs. four) from the previous round led to cumulative improvements in flight distance. The authors concluded that having access to a larger number of variants is taxing the working memory, which could lead to lower copying fidelity. Having a higher innovation rate similarly reduces the social learning success of one's social connections. The reduction in overall social learning probability with the number of options available is akin to the well-documented phenomenon of ‘choice overload’ in consumer psychology (Chernev et al., 2015; Iyengar & Lepper, 2000; Scheibehenne et al., 2010): having too many choices for a consumer good can result in a number of counterintuitive effects, including a reduction in the probability of purchasing anything at all. In our case, this pattern flows directly from the requirement that in order to socially acquire a trait, individuals have to be exposed to it multiple times. This means that the probability of social learning is non-linear in the frequency of the trait in an individual's social neighbourhood and decreasing in the number of skills that are available for observation.

Because of this conflict between group-level payoffs and individual-level selection, high-proficiency culture and the social structure that maintains it can be considered a public good: maintaining the social learning infrastructure for high-proficiency culture requires individuals to make more connections and innovate less than what would maximise their own payoffs. As in other public goods, groups can counteract socially harmful individual incentives. For example, Ache hunter–gatherers maintained a high yearly interaction rate and high number of total connections for their population size compared with the Hadza hunter–gatherers, in part by organising inter-band club fight rituals (Hill et al., 2014). Likewise, in Southern India, participating in religious rituals results in denser connections (Power, 2018). Our results provide a novel hypothesis for the evolution of rituals and social norms that promote social connections can enforce connectivity, cultural convergence and the resulting high proficiency, which can have an advantage in competition with other groups. One further factor modulating the connectivity of the learning network might be the traits themselves: traits used in cooperative foraging or social rituals will necessarily have more connected learning networks than traits that are mostly for personal or household use, such as knowledge about medicinal plants (Salali et al., 2016).

Likewise, as we find too much individual innovation ultimately is detrimental to high proficiency culture, we might expect norms that enforce social learning at the expense of individual innovation, even if there is no inherent difference between the traits that are socially transmitted. Such normative behaviour discouraging individual innovation has been described, for example, for the transmission of pottery skills among three ethnic groups (Dii, Duupa, and Doayo) in Cameroon (Wallaert-Pêtre, 2001). Here, practitioners form tight communities that limit access to knowledge and reject departures from socially admitted norms. This leads to highly conserved methods and products, which are often observed in the context of formal apprenticeships (see e.g. Lancy, 2012). Similarly, Buckley and Boudot (2017) provide an example of loom and weaving technology in Southeast Asia, where knowledge is primarily transmitted from mothers to daughters over a long apprenticeship, which similarly leads to relatively low rates of innovation.

Another potential solution to the problem of maintaining a specialised and high-proficiency cumulative culture is role specialisation. Our model considers a simple, undifferentiated population; this might be an ancestral state for human populations but the tensions identified in our model are expected to impose strong selection for differentiation of roles with respect to social learning. Specifically, high effective connectivity with respect to social transmission of traits can also be achieved if learning happens mostly through a subset of individuals that specialise in teaching social information. These individuals could also invest into making transmission more efficient by directed demonstrations or focusing attention (Ventura & Akcay, 2022). This is an additional selective advantage for the evolution of teaching, in addition to ensuring transmission fidelity (Castro & Toro, 2014).

Our results with heterogeneous traits and environmental turnover highlight an additional dimension of complexity in the coevolution of cumulative culture and social structure. Environmental turnover rate emerges as an important modulator of the type of network structure and cumulative culture, with rapid environmental turnover favouring disconnected graphs, larger repertoires and low proficiency. These results are in line with previous results (Deffner & Kandler, 2019; Kolodny et al., 2015) showing that stable environments lead to more specialised kinds of culture. Consistent with our predictions, Kalan et al. (2020) found that chimpanzee groups living in more variable environments exhibited greater trait diversity. On the other hand, heterogeneity in payoffs at a given point in time has contrasting effects depending on the turnover rate: at low turnover rates, where payoffs update infrequently, larger variance favours populations that find the high payoff traits and increase proficiency in them by becoming well connected. However, at high turnover rates it becomes likely that a high payoff trait will no longer be high payoff by the time proficiency is accumulated, which forecloses this route to cultural adaptation. Therefore, individuals are selected to increase their repertoire size to try to maximise the probability of learning at least some high-payoff traits. Notably, in most cases, populations still visit both the highly connected, high-proficiency state and the sparsely connected, broad-repertoire state, indicating that environmental heterogeneity and turnover modulate but do not entirely eliminate the tensions described above.

Our model highlights how the interaction of individual innovation, social learning and connectivity creates emergent constraints on the evolution of cumulative culture. This has implications for the much-discussed topic of how cultural complexity relates to population size. We find that cultural complexity does not keep increasing with population size (Figure S13) because further increases in complexity run into the limits of social learning at the individual level. This effect was also observed in a previous model by Mesoudi (2011) where social learning and individual innovation were both assumed to incur a direct cost, putting a limit to the amount individuals can learn. These results suggest that a population size effect on cumulative culture will be limited in the absence of further innovation in learning technology (as discussed above) that can ameliorate the trade-offs in social learning. We further show here that the effect of population size on cumulative culture is contingent on the environment: the increase in cultural complexity in the form of increased proficiency requires that the environment changes relatively slowly. Otherwise, what is profitable changes before any proficiency can be accumulated through cultural transmission, and population size has no effect on the cultural complexity.

Overall, our model points to previously unappreciated tensions between different pathways to cultural adaptation in groups and the incentives at the group and individual level that arise from the coevolution of interaction networks and cumulative culture. Resolving these tensions in different ecological settings probably played an important role in human social evolution and our ability to do so might be one reason for the unique success of our species.

## Data Availability

n/a

## Supplementary material

For supplementary material accompanying this paper visit https://doi.org/10.1017/ehs.2023.21. click here to view supplementary material

## Research transparency and reproducibility

To reproduce the simulations and figures presented in this paper use the code that is provided at https://github.com/marcosmolla/pathways_cultural_adaptation.
