# Supplementary material for: Pathways to cultural adaptation: the coevolution of cumulative culture and social networks
Source: Evol Hum Sci. 2023 Aug 25;5:e26. doi: 10.1017/ehs.2023.21 (PMC10565192; doi:10.1017/ehs.2023.21)
Supplement: Supplementary file 1 [file ehssup.zip › S2513843X2300021Xsup002.pdf]

Supplementary Material

Pathways to cultural adaptation: the coevolution of cumulative culture and social networks

Marco Smolla and Erol Akçay

S1 Payoff distribution

To simulate heterogeneous trait utilities, we draw random values from a lognormal distribution. Examples for relevant values in the main text are shown in Fig. S1.

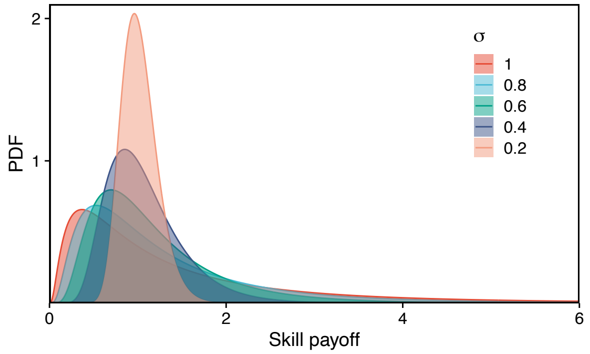

Figure S1. Probability density function for the different payoff variances used in the main text. Values are drawn from a lognormal distribution, Lognormal(μ, σ^2) with $\mu = 0$ and $\sigma^2 \in \{0.2, 0.4, 0.6, 0.8, 1\}$. As σ^2 increases, the distributions becomes more skewed. Payoffs drawn from these distributions have a mean of $\mu = \{1, 1.1, 1.2, 1.4, 1.6\}$ and a variance of var $= \{0.04, 0.20, 0.62, 1.7, 4.66\}$.

S2 Fixed homogeneous environments

S2.1 Cultural selection on complex graphs

Next, we let the linking parameters evolve in fixed and homogeneous environments. We observe which networks emerge, and how this affects individual and population-level culture.

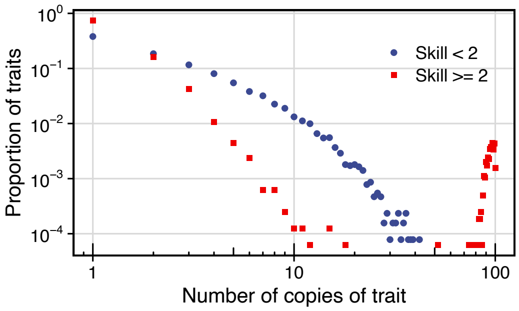

Figure S2. Trait frequency distributions differ between high proficiency and large repertoire populations. Populations with low average skill have in general more traits with intermediate frequency, whereas those with higher skill have a few traits with very high frequency in the population. Simulations with $\alpha = 0.01$ and $\beta = 1$, $N = 100$, $M = 500$, running for 5,000 generations, $\tau = 0$ and $\sigma = 0$.

S2.2 Robustness checks

We ran several robustness checks. First, to test that selection is acting here, we remove selection. When the two linking parameters are left to drift, we do not observe these two kinds of populations. Instead we find networks that are mostly connected, and populations with high proficiency but none with large repertoires (Fig. S3). Next, we ran simulations with fixed linking parameters to test that the differences in repertoire size and proficiency are, in fact, a result of the social network (Fig. S4). We observe patterns that correspond with the above results. To ensure that the observed patterns do not rely on the complex networks that we simulate here but are a general phenomenon, we simulated learning on random graphs with different degree. Consistent with the results above we find that repertoire size and count of unique traits decrease and proficiency and payoff increase as degree increases (Fig. S5,S6). Finally, we tested different linking parameter mutation rates, m (Fig. S7).

Neutral selection For the neutral case (Fig. S3), we see that p_{n} and p_{r} spread equally in all directions. This results in networks that are mostly connected (average weighted component size equals N). We also see that there is a proficiency and repertoire size trade-off, as we would expect. Populations with connected networks also have on average high skill proficiency, albeit smaller repertoires and fewer overall known traits.

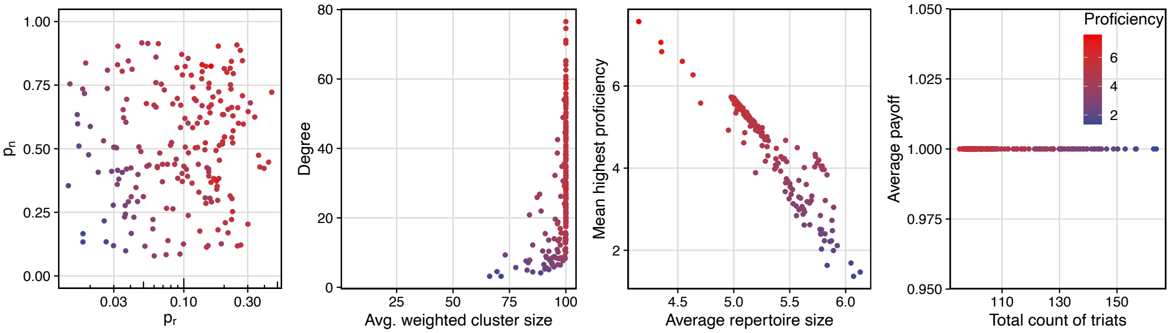

Figure S3. The neutral evolution of linking parameters p_{n} and p_{r} results in mostly connected graphs (large component size). In all plots colour indicates mean highest proficiency of a population. We find that populations with larger average degree reach higher proficiency. Populations with these networks also know on average fewer different traits. There is a clear negative relationship between proficiency and repertoire size, which is based on the trade-off during the limited number of learning turns: learn a new trait versus improve a known trait (see also Smolla and Akçay (2019)). Simulations with $\alpha = 0.02$ and $\beta = 1$, $N = 100$, $M = 500$, running for 5,000 generations, $\tau = 0$ and $\sigma = 0$, averaged over 200 repetitions.

Cultural selection on complex graphs with fixed linking parameters We find two repertoire types with according network shapes when we fix linking parameters in simulations (so, there is still selection on survival, but linking parameters do not change over time). High p_{n} as well as high p_{r} lead to connected graphs, which result in a low overall skill count and small average repertoire sizes but higher skill level.

Cultural selection on random graphs We run the learning algorithm described in the Methods section on random graphs (Fig. S5 and S6) to provide another reference point for the

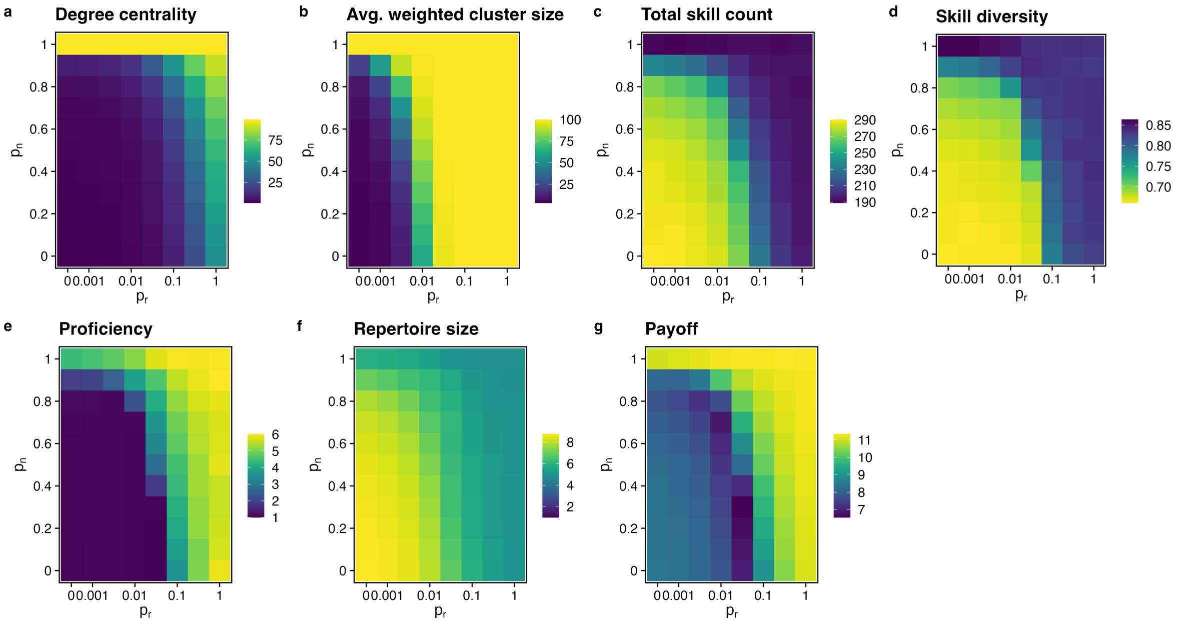

Figure S4. Linking parameters affect whether populations rely on high proficiency or large repertoires. The space that the two linking parameters p_n and p_r span can be separated into two areas. First, populations with low p_n and low p_r form networks with lower mean degree centrality (a) that are largely unconnected (b). They have the most diverse trait knowledge (c), which also means that they have the lowest proficiency (e) albeit the largest individual repertoires (f). The second zone is for high p_n and/or high p_r, where we find connected graphs with high mean degree centrality. Here, proficiency is high and repertoires are small. Overall, populations in this zone know a smaller number of traits in total, however, they have on average a higher payoff then populations in the other zone. Simulations with $\alpha = 0.02$ and $\beta = 1$, $N = 100$, $M = 500$, running for 5,000 generations, $\tau = 0$ and $\sigma = 0$, averaged over 200 repetitions.

simulation with complex networks in the main text. We simulate different population sizes as well as different average number of neighbourhoods an individual is sampling from (so in principle these are random graphs).

For the Proficiency/Repertoire size space, we find two types: low and high proficiency, whereby the latter has a slightly smaller repertoire size on average. Also, larger networks have on average higher proficiency and smaller repertoires when compared to smaller networks. Though, larger networks on average have a larger number of total traits.

Different mutation rates In a previous version of the model we tested higher values for mutation which yielded qualitative similar results (Smolla and Akçay, 2019). We tested different values of m here as well and document the results in Fig. S7.

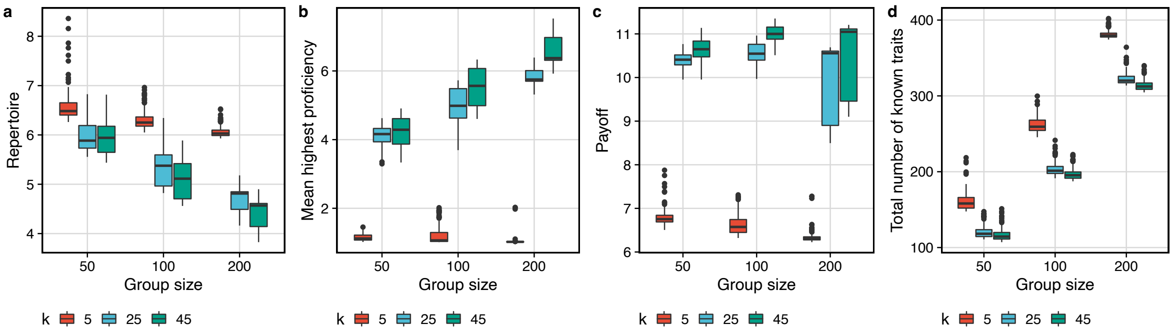

Figure S5. Population size as well as mean degree centrality affect individual and population-level culture. Individuals in larger populations have on average smaller repertoires and higher proficiency. Larger populations also know on average more different traits than smaller populations. Across all population sizes, individuals in networks with smaller k have larger repertoires and lower proficiency, and yield a lower payoff. Populations with smaller degree also know on average more different traits than those with a higher degree. Payoffs on average increase with population size, at least for high mean degree centrality. However, for low degree payoff decrease for larger populations. This is because of the larger number of known traits, which causes there to be a very high trait diversity in any individual's neighbourhood which ultimately leads to many individuals learning even less (which we can see in the smaller average repertoire and the lower average proficiency of the large groups). We simulate populations with complete graphs but allow individuals only to learn from k random neighbours.Simulations with $\alpha = 0.02$ and $\beta = 1$, $N \in \{50, 100, 200\}$, $M = 500$, running for 5,000 generations, $\tau = 0$ and $\sigma = 0$, with 200 repetitions for each set of parameters.

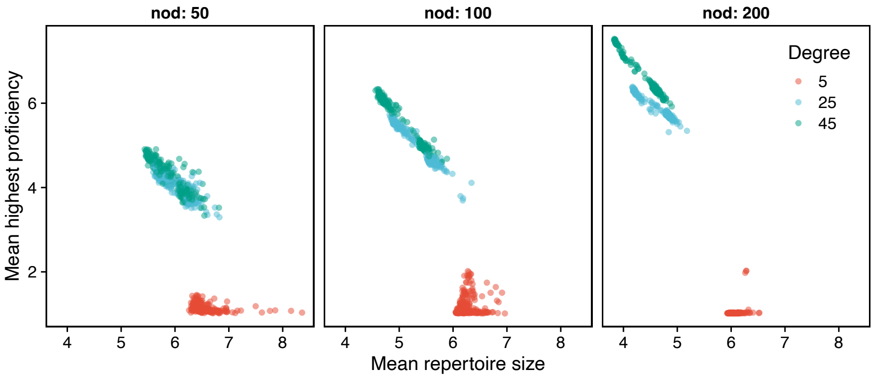

Figure S6. Connectivity affects repertoire type. Populations with higher average degree rely on high proficiency, whereas populations with sparser networks have low proficiency but slightly larger repertoires. As population size increases, so does the average highest proficiency reached in populations with intermediate and high mean degree centrality. Proficiency learning benefits from more frequent innovation in larger populations and cultural convergence due to high connectivity. In contrast, larger populations with sparse networks do not achieve larger repertoires. Despite harbouring more unique cultural traits (Fig. S5d), there is still a limit to the diversity in an individual's neighbourhood for successful social learning. In fact, the larger number of cultural traits in the population leads to a slightly reduced repertoire size and consequentially smaller average payoff (Fig. S5c). Data from simulation in Fig. S5.

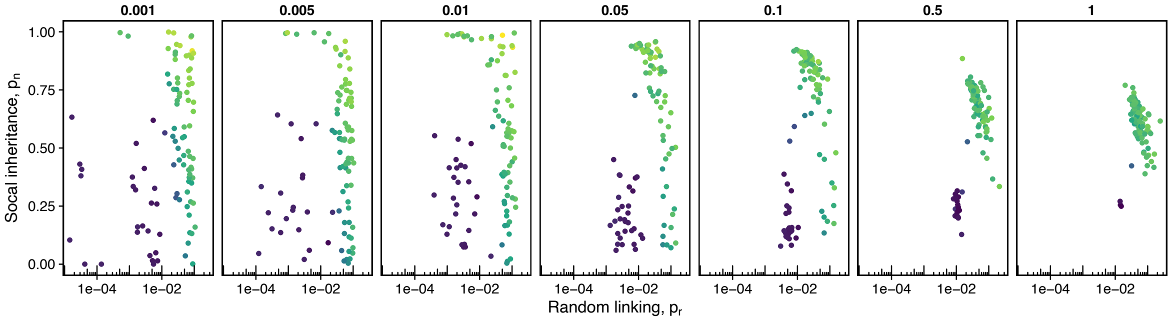

Figure S7. Changing mutation rate, m**.** For large mutation rates ($m > .05$) populations are less likely to reach the high payoff state of high p_{n} as the strong mutation will frequently push populations away from this boundary. In all plots colour indicates mean payoff (dark blue indicating low payoffs and yellow high payoffs). Simulations with $\alpha = 0.0$ and $\beta = 1$, $N = 100$, $M = 500$, running for 5,000 generations, $\tau = 0$ and $\sigma = 0$, averaged over 200 repetitions. The values of m are indicated above each plot.

Table S1. Results of Markov chain analysis of cycling behaviour. Using data from simulations with different population sizes N (all with homogeneous environments, $\tau = 0$, $\sigma = 0$, running for 40,000 generations, with $\alpha = 0.01$ and $\beta = 1$), we first counted the number of times populations switched between the high and low payoff state. That is, we counted the number of times when a population's mean payoff exceeded 60% of the maximum payoff from across all simulations, or fell below this threshold (we tested different thresholds but found this threshold to best separate the high and low payoff simulations). Next, we pooled the data from all 100 simulations and calculated the probability for a switch to occur in a single population from one generation to the next. We used these probabilities to construct a transition matrix for a Markov process and calculated the stationary distribution of both states (high and low payoff).

	$N = 50$	$N = 100$	$N = 500$
Prob. switch Up to Down	0.01013	0.000114	2.10e-05
Prob. switch Down to Up	0.01018	0.000141	4.73e-05
Stationary proportion high	0.5	0.55	0.69
Stationary proportion low	0.5	0.45	0.31

S2.3 Cycling

Figure S8 shows trajectories of five example populations at different time points, illustrating the cycling phenomenon described in the main text. Figure S9 provides a closer look at the transitions between high and low payoff states.

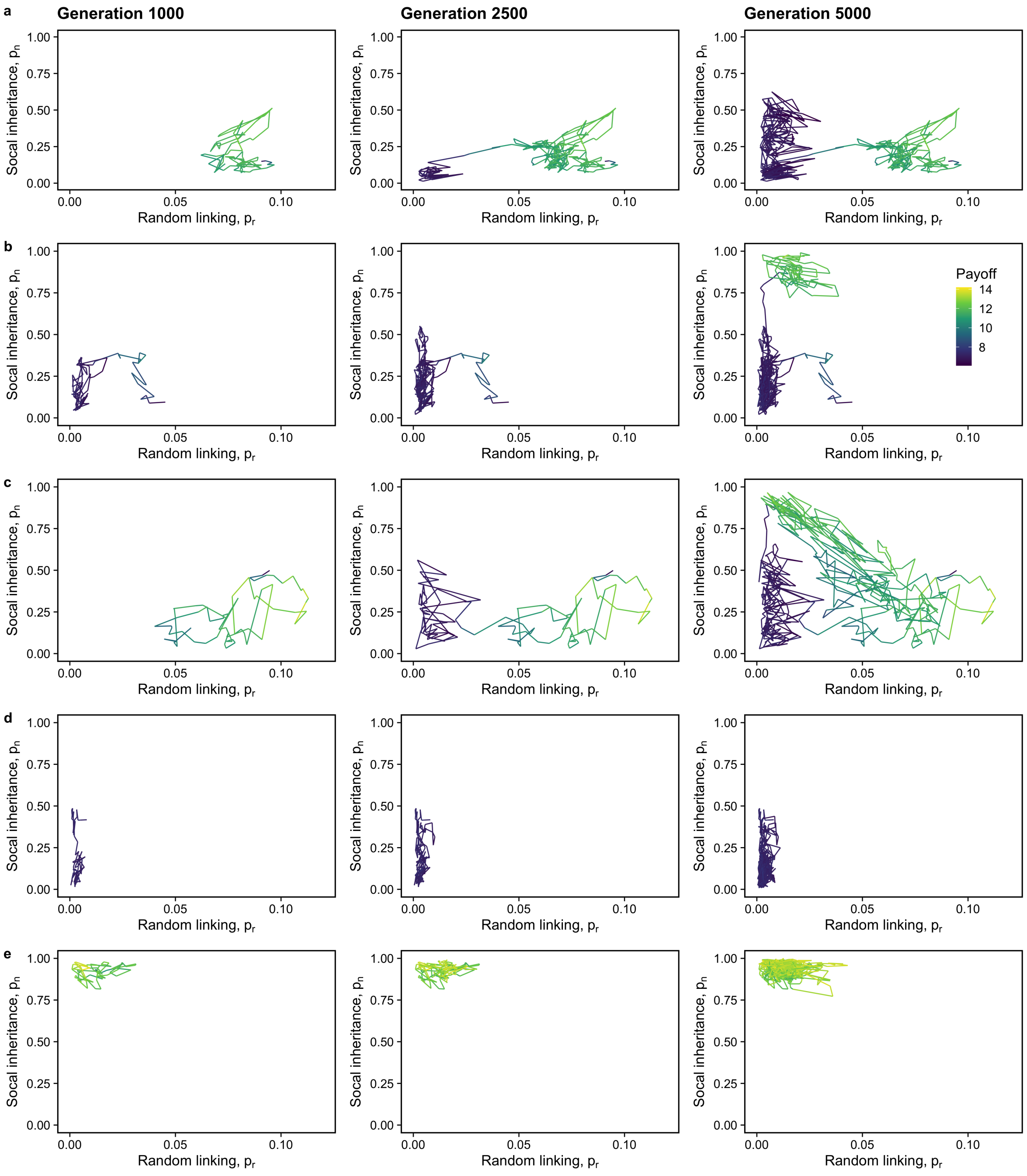

Figure S8. Examples of population connectivity and fitness through time. Data shown are from the same simulations as those in Fig. 2 but for five individual populations (a-e) at three different times throughout the simulation (at 1000, 2500, and 5000 generations). Some populations begin in the high payoff state (high p_n or p_r) and eventually 'fall' into the fitness valley (low p_n, p_r, a-c). Other begin in the low (d) or high (e) payoff state and remain there till the end of the simulation, or even 'escape' from the fitness valley (b,c).

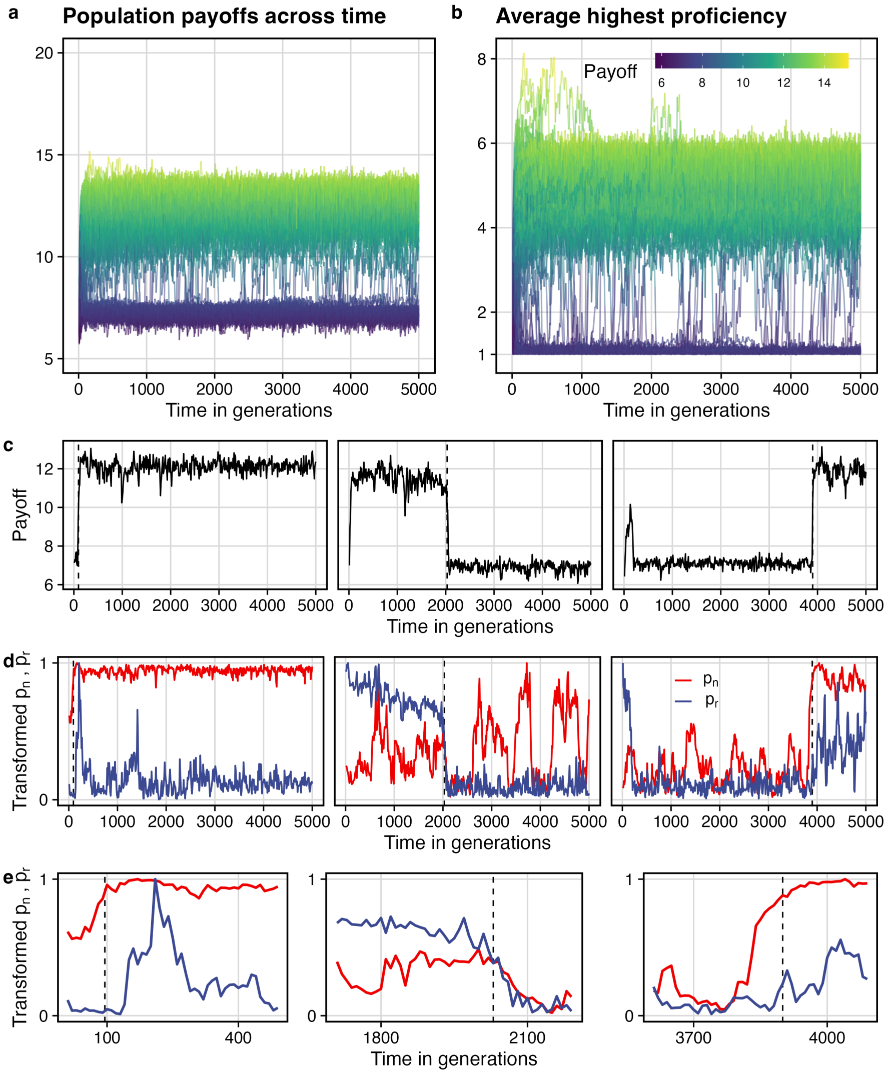

Figure S9. A closer look at transitions between high- and low-payoff states. Panels a and b depict the time-trajectory of an ensemble of simulations that show that they quickly reach a steady state. Panels (c) depicts three individual simulations that provide examples of switches between the low and high-payoff states, with the corresponding transitions in the linking traits (normalised, so that 1 represents the highest observed value) depicted in panels below (d). As can be seen in the close-ups in panels (e), a transition to a high-payoff state begins with an increase in p_{n} and a transition to a low payoff state with a decrease in p_{r}. Simulations with $\alpha = 0.02$ and $\beta = 1$, $N = 100$, $M = 500$, running for 5,000 generations, $\tau = 0$ and $\sigma = 0$.

S2.4 Simple contagion based social learning

Here, we show that the social learning model that requires repeated exposures is necessary to get the two distinct pathways. The repeated exposures requirement underlies our assumption that the probability of socially learning a trait is proportional to the square of its frequency in an individual's neighbourhood. Figure S10 shows results of simulations in homogeneous, fixed environments where instead we assume social learning probability is proportional to the frequency of the trait in the neighbourhood. It shows that populations are selected to be always well connected, accumulate very high repertoires compared to the repeated exposure model and low proficiencies. The latter happens because in the absence of coordination on a few traits, more innovations that improve the proficiency get lost before they can get transmitted. This shows that the conflict between individual selection and group selection is absent with single exposure social learning.

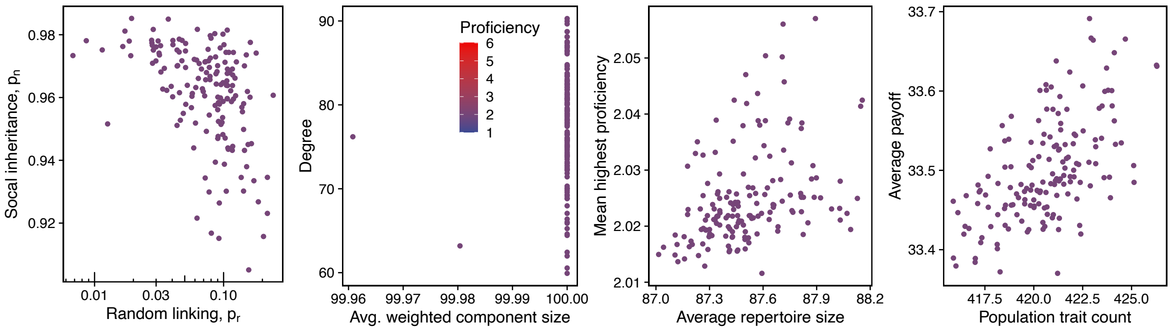

Figure S10. Results from simulations in a homogeneous, constant environment where social learning requires a single exposure. Here, we assume that the probability of socially learning a trait from connections is proportional to its frequency in an individuals neighbourhood (as opposed to proportional to the square of the frequency as in our main results). This corresponds to the assumption that learning can happen with a single exposure. These figures show that the repeated exposure assumption is necessary for the main results we describe in the main text. Specifically, when social learning happens with a single exposure, there is no individual selection for reduced connectivity and all populations are selected to be extremely connected (note that the range on the axes in the first two panels correspond to highly connected networks), have very large repertoire sizes, and display no trade-off between repertoire size and proficiency (which is generally low, since populations cannot converge on the same traits). Figure S18 shows the equivalent results in heterogeneous and changing environments. In contrast to the social learning described in the main text, here, in a single copying attempt the individual is sampling randomly from the traits in its neighbourhood weighted by the frequency of the trait ($p_{i,t}$) and is successfully acquiring the trait with probability β.

S3 Adding environmental heterogeneity to cultural selection on complex graphs

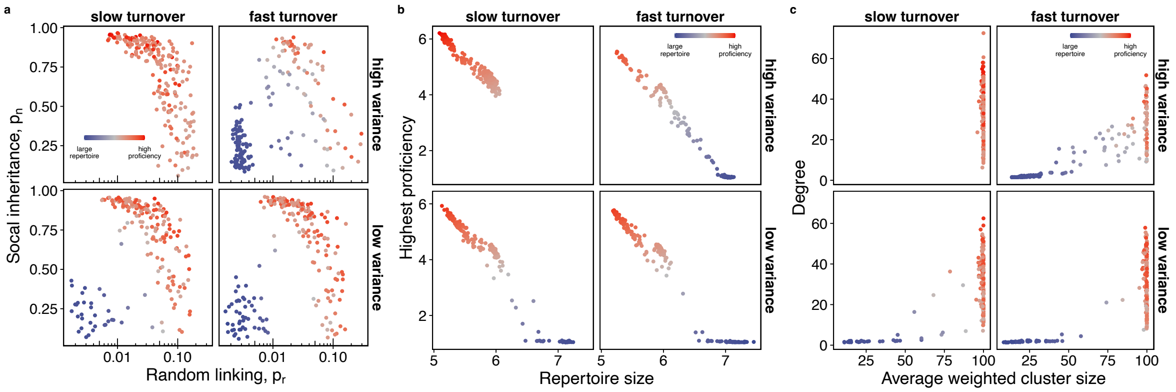

Figure S11. Environmental effects on the evolution of linking parameters. The equivalent of Figure 1 in the main text for four environmental conditions. Colour scale is based on the following data transformation of average repertoire size R and highest proficiency L: $L/\max(L) - R/\max(R)$. Simulations with $\alpha = 0.01$ and $\beta = 1$, $N = 100$, $M = 500$, running for 5,000 generations, $\tau \in \{10^{-3}, 1\}$ and $\sigma = \{0.2, 1\}$.

S3.1 Varying individual and social learning success probability

Whether either of the strategies is dominant is not only affected by the environment, but also very much by the frequency of innovation and the reliability of social learning.

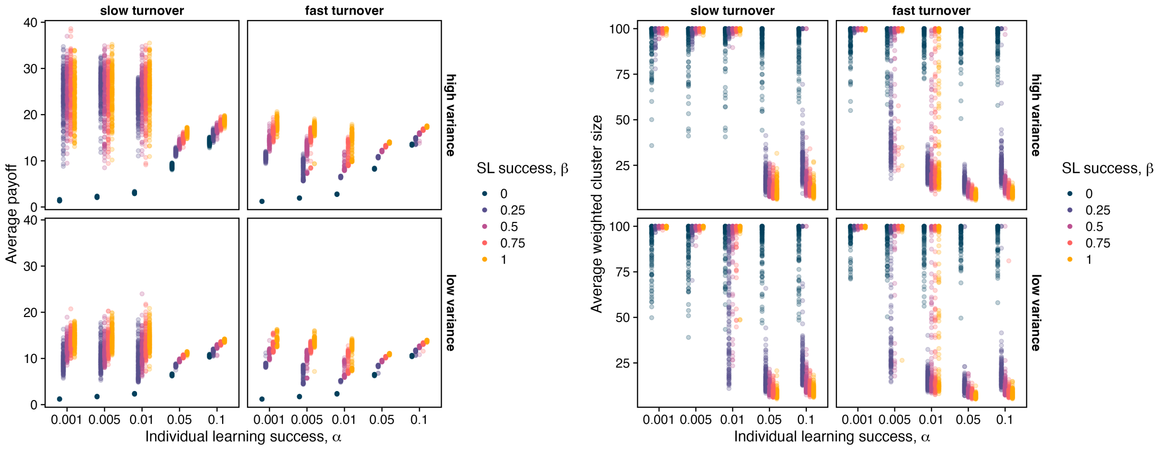

Figure S12. The rate of innovation and social learning affects payoffs in heterogenous environments. The average payoff (a) and average connected component size (b) as a function of social learning rate, for different rates of individual innovation rates. As in the homogenous environment case, in the absence of social learning, we find that increasing innovation always increases payoffs. However, for non-zero social learning success, we find that intermediate values of innovation yield lower payoffs compared to high and low innovation rates. Simulations with $N = 100$, $M = 500$, running for 5,000 generations, $\tau \in \{10^{-3}, 1\}$ and $\sigma = \{0.4, 1\}$.

S3.2 Varying population size

We investigated the effect of population size on coevolution of cumulative culture and social network structure in heterogeneous environments. Figure S13 depicts two of the diagnostic outcomes, average proficiency and connected component size (relative to population size) against population size for different environmental conditions. It shows that only environments with slow turnover produce high proficiency populations, and only when the variance amongst traits is the high proficiency state is dominant. For slow turnover populations, higher population size allows higher proficiency to be built up. However, the proficiency can only go up to a point: with these parameters, population sizes beyond 100 do not cause further increases in the mean proficiency in the high variance slow turnover condition. A more detailed look in figures S14 and S14 corroborate these patterns.

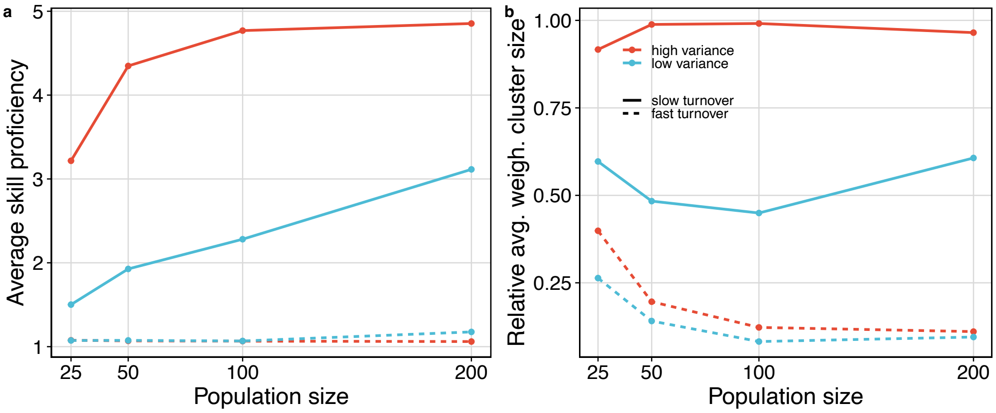

Figure S13. Effect of population size on the coevolution of cumulative culture and social network structure in heterogeneous environments. Each line depicts one prototypical environmental condition: solid lines for slow turnover, dashed for fast; red lines for high variance in payoffs between traits, and blue for low variance. Only slow turnover environments produce high proficiency populations; in these environments, increasing population size allows higher proficiency to be built up. Simulations with $\alpha = 0.02$ and $\beta = 1$, $N = 100$, $M = 500$, running for 5,000 generations, $\tau \in \{10^{-3}, 1\}$ and $\sigma = \{0.4, 1\}$.

S3.3 Fertility selection

In the main text, we assume that the cultural traits affect the probability of the individuals to be selected to die at each time step (mortality selection). An alternative we implemented in our previous work (Smolla and Akçay, 2019) is that the cultural traits affect the probability of being selected to reproduce. To check whether implementing selection at the mortality stage vs. reproduction affects our main results, we ran our simulations with selection at the reproduction stage. Specifically, in each time step, one individual is selected at random to die, but the individual to reproduce is selected with probability proportional to $M(W_i)$ as defined in the methods. Figure S16 shows the equivalent of Figure 1 in the main text. It shows that our results are not affected by mortality vs. fertility selection. Specifically, we again observe the same two distinct population states (low connectivity, broad repertoire, low proficiency and payoff vs. high connectivity, narrower repertoire, high proficiency and payoff). Moreover, we also see the cycling between these states, driven by the exact same patterns in payoffs depicted in Figures 2 and 3.

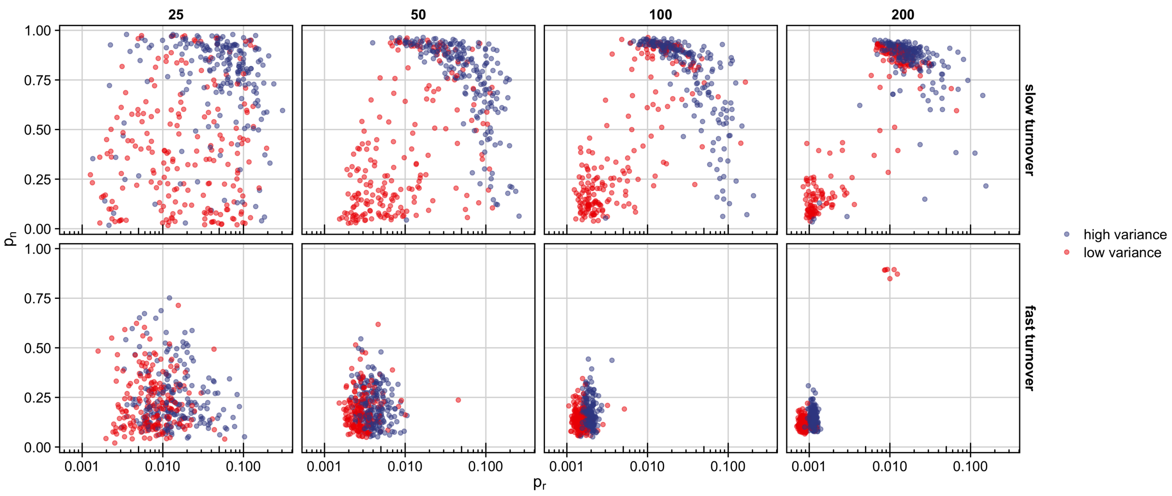

Figure S14. More detailed look at the populations' linking traits at different population sizes across environments. In slow turnover environments, increasing population size tends to favour more connected populations, whereas in fast turnover environments, increasing population size has a much weaker effect. For slow turnover, population size effect is stronger with high variance between traits, while for fast turnover, the population size effect is only apparent with low variance. Simulations with $\alpha = 0.02$ and $\beta = 1$, $N = 100$, $M = 500$, running for 5,000 generations, $\tau \in \{10^{-3}, 1\}$ and $\sigma = \{0.4, 1\}$.

S3.4 No saturating payoffs

As described in the methods, we assume that the mortality probabilities are functions of not directly the payoff W_i, but a saturating function of it $M(W_i)$, which we assume has a Michealis-Menten form. We assume a relatively large half-rate constant ($K = 50$) which means that for most parameter regimes, $M(W_i)$ is closer to linear in W_i, as the latter is not close to the half-rate constant. As explained in the methods, we made this assumption to guard against the possibility that in heterogeneous environments with large payoff variance between traits, a single "lucky" innovation with very high results might introduce unrealistic amount of strong selection. To check whether this assumption is consequential for our base results, we ran our simulations in homogeneous environments assuming selection (probability of mortality in a given time step) is directly proportional (to the inverse of) W_i, instead of a saturating function of it. Figure S17 depicts the equivalent of Figure 1 in the main text, and shows that this assumption is not consequential for our main result.

S3.5 Simple contagion based social learning in heterogeneous environments

Figure S18 shows the equivalent of S10 in different heterogeneous and changing environments. These simulations again assume that social learning probability is proportional to the frequency of the trait in the neighbourhood rather than the square of the frequency. It shows that all of the results in homogeneous environments carry over to heterogeneous environments, with slight changes as expected due to the payoff variation between traits and turnover rate. Specifically, we again do not find the conflict between group payoff and individual fitness, and therefore no cycling.

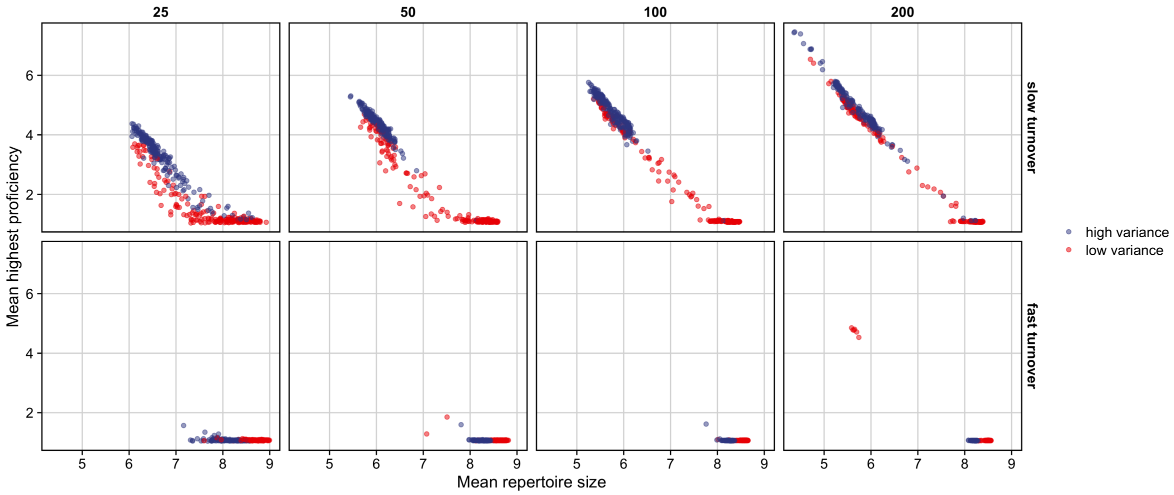

Figure S15. Proficiency and repertoire size with population size across environments. The mean proficiency and repertoire size for the same populations as in Figure 2. Increasing population size in slow changing environments favours increased proficiency (especially with high variance), which in fast changing environments it has relatively little effect, and that only under low variance. Simulations with $\alpha = 0.02$ and $\beta = 1$, $N = 100$, $M = 500$, running for 5,000 generations, $\tau \in \{10^{-3}, 1\}$ and $\sigma = \{0.4, 1\}$.

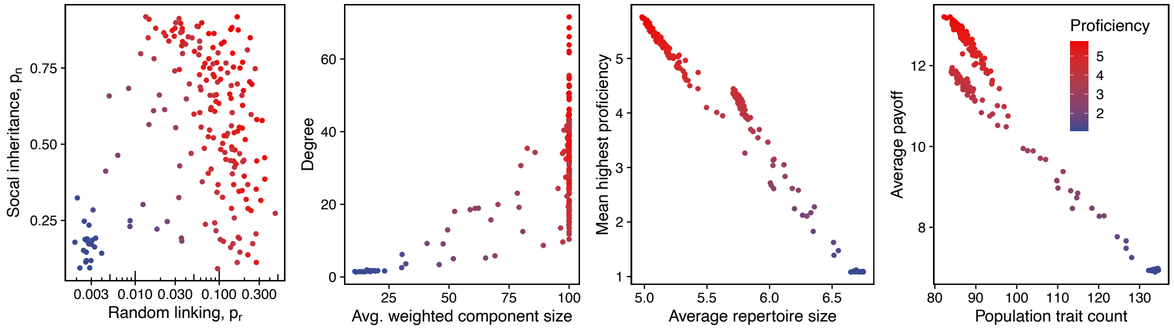

Figure S16. Results with fertility selection in homogeneous environments. The equivalent of Figure 1 in the main text, but with selection due to cultural traits on fertility instead of mortality. Specifically, in these simulations, mortality is random but probability is getting selected to reproduce is proportional to $M(W_i)$ as described in the methods. All our results in homogeneous environments carry over to fertility selection, including the two pathways to cultural adaptation and cycling between them. Simulations with $\alpha = 0.01$ and $\beta = 1$, $N = 100$, $M = 500$, running for 10,000 generations, $\tau = 0$ and $\sigma = 0$, 200 replicates.

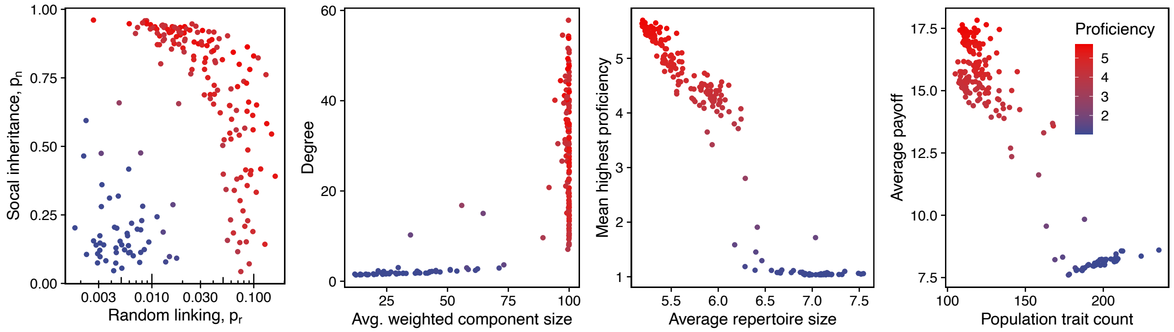

Figure S17. Results without saturating fitness values. The equivalent of Figure 1 in the main text, but with fitness simply equal to payoff W_i, i.e., without the saturating Michealis-Menten function. All our results from the main text again carry over to the case without the saturating fitness function. Simulations with $\alpha = 0.01$ and $\beta = 1$, $N = 100$, $M = 500$, running for 5,000 generations, $\tau = 0$ and $\sigma = 0$, 200 repetitions.

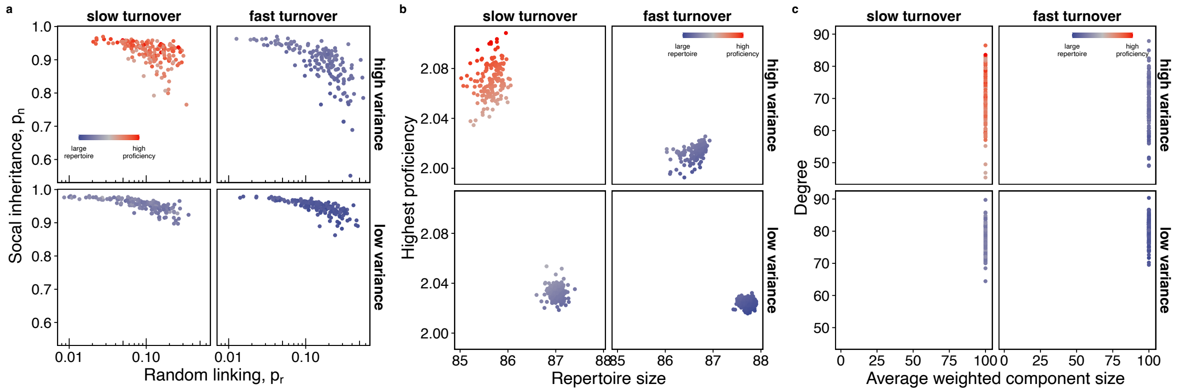

Figure S18. Results from simulations where social learning requires only a single exposure. Compared to the results from the main text (where we assume social learning requires multiple exposures), we do not find the two different pathways (dense networks with high proficiency and small repertoire vs. sparse networks with large repertoires and low proficiency). Therefore, we also do not find the cycling behaviour that we describe in the main text results. Instead, we find that dense network evolve (across all tested environments) and that proficiency remains low but repertoires are substantially larger. For simple contagion the probability to acquire a specific trait is $\beta p_{i,t}$, whereas for complex contagion it is $\beta p_{i,t}^2$. This makes acquiring traits with single exposure much more likely, which leads to the larger repertoires. On the other hand, having a large repertoire and a limited number of learning turns limits the probability to successfully 'level up' in any trait, which causes proficiency to remain low. Colour scale is based on the following data transformation of average repertoire size R and highest proficiency L: $L/\max(L) - R/\max(R)$. Simulations with $\alpha = 0.01$ and $\beta = 1$, $N = 100$, $M = 500$, running for 5,000 generations, $\tau \in \{10^{-3}, 1\}$ and $\sigma = \{0.2, 1\}$, each with 160 repetitions.
