# Supplementary material for: Pathways to cultural adaptation: the coevolution of cumulative culture and social networks
Source: Evol Hum Sci. 2023 Aug 25;5:e26. doi: 10.1017/ehs.2023.21 (PMC10565192; doi:10.1017/ehs.2023.21)
Supplement: Supplementary file 1 [file ehssup.zip › S2513843X2300021Xsup001.zip!/images/220927_2_neutral_scatter_neutral_all0_WD70UTK.pdf]

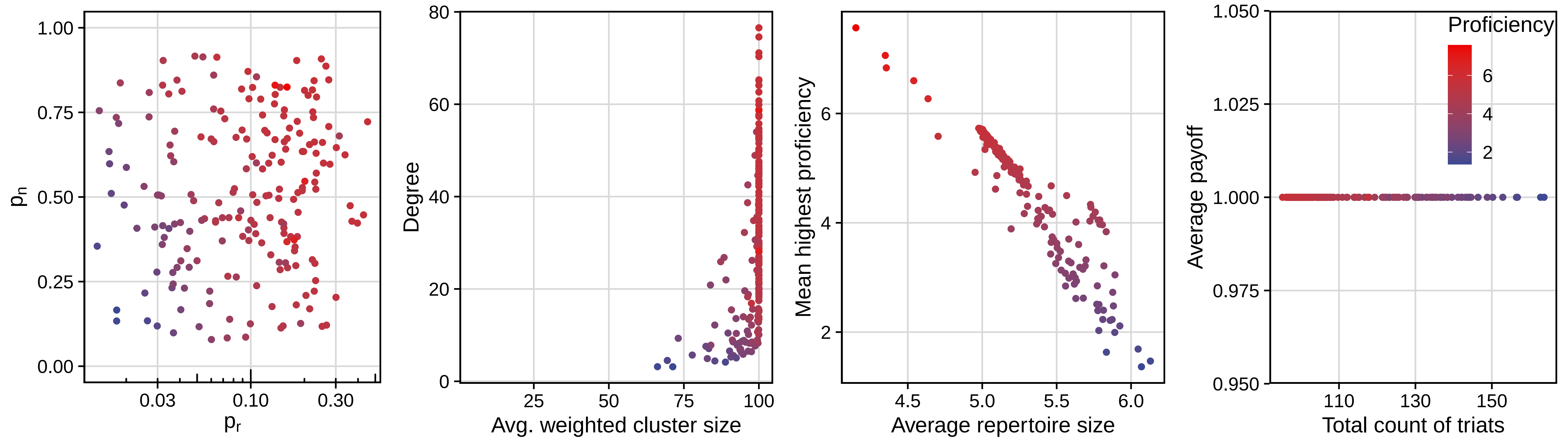

1.00
0.75
0.50
0.25
0.00
p_n
0.03
0.10
0.30
p_r
80
60
40
20
0
Degree
25
50
75
100
Avg. weighted cluster size
6
4
2
Mean highest proficiency
4.5
5.0
5.5
6.0
Average repertoire size
1.050
1.025
1.000
0.975
0.950
Average payoff
110
130
150
Total count of triats
Proficiency
6
4
2
