# Supplementary material for: Pathways to cultural adaptation: the coevolution of cumulative culture and social networks
Source: Evol Hum Sci. 2023 Aug 25;5:e26. doi: 10.1017/ehs.2023.21 (PMC10565192; doi:10.1017/ehs.2023.21)
Supplement: Supplementary file 1 [file ehssup.zip › S2513843X2300021Xsup001.zip!/images/220928_5_completeGraph_learnig_scatter_neutral_all0_BK31PVU.pdf]

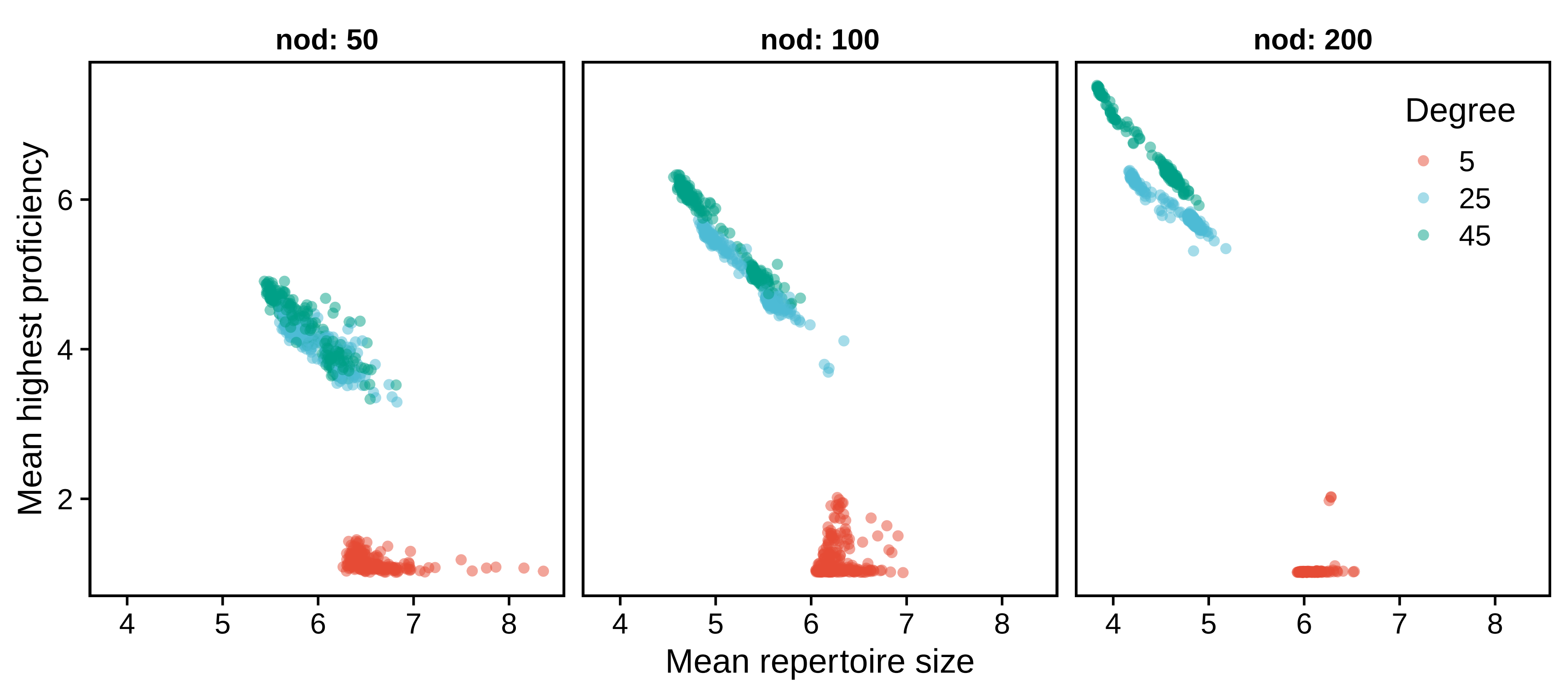

nod: 50
nod: 100
nod: 200
Mean highest proficiency
Mean repertoire size
2
4
6
4
5
6
7
8
Degree
5
25
45
