# Supplementary material for: Pathways to cultural adaptation: the coevolution of cumulative culture and social networks
Source: Evol Hum Sci. 2023 Aug 25;5:e26. doi: 10.1017/ehs.2023.21 (PMC10565192; doi:10.1017/ehs.2023.21)
Supplement: Supplementary file 1 [file ehssup.zip › S2513843X2300021Xsup001.zip!/images/220928_5_completeGraph_learnig_overview_PJ55LEQ.pdf]

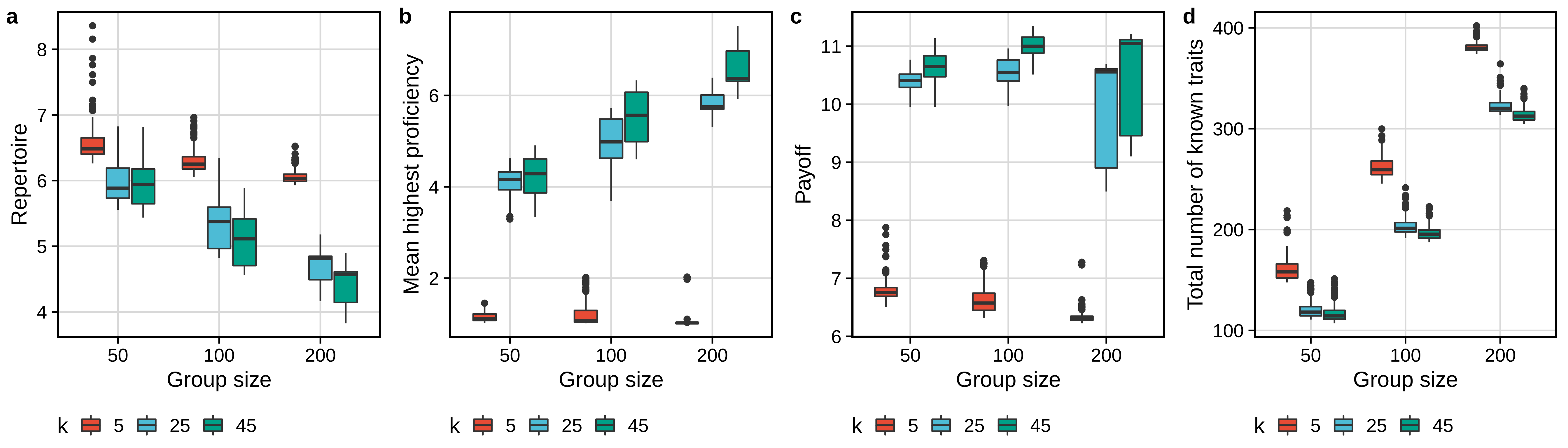

a
Repertoire
Group size
b
Mean highest proficiency
Group size
c
Payoff
Group size
d
Total number of known traits
Group size
k 5 25 45
