# Supplementary material for: Pathways to cultural adaptation: the coevolution of cumulative culture and social networks
Source: Evol Hum Sci. 2023 Aug 25;5:e26. doi: 10.1017/ehs.2023.21 (PMC10565192; doi:10.1017/ehs.2023.21)
Supplement: Supplementary file 1 [file ehssup.zip › S2513843X2300021Xsup001.zip!/images/230220_2_test_simpleContagion_scatter_all0_CP26PDF.pdf]

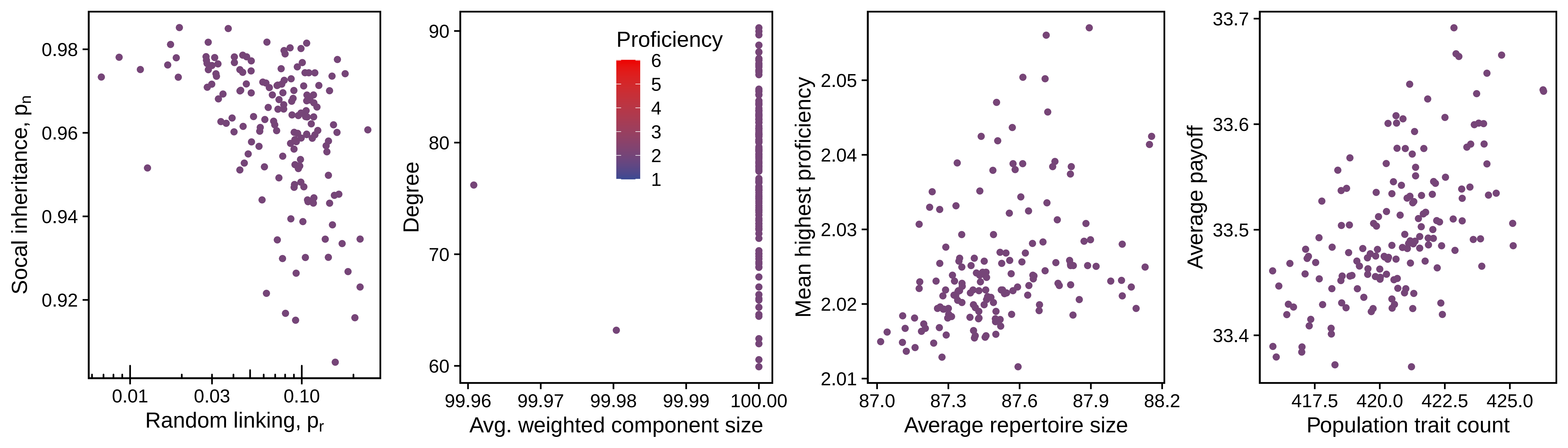

Socal inheritance, p_n
0.98
0.96
0.94
0.92
0.01
0.03
0.10
Random linking, p_r
Proficiency
6
5
4
3
2
1
Degree
90
80
70
60
99.96
99.97
99.98
99.99
100.00
Avg. weighted component size
Mean highest proficiency
2.05
2.04
2.03
2.02
2.01
87.0
87.3
87.6
87.9
88.2
Average repertoire size
Average payoff
33.7
33.6
33.5
33.4
417.5
420.0
422.5
425.0
Population trait count
