# Supplementary material for: Pathways to cultural adaptation: the coevolution of cumulative culture and social networks
Source: Evol Hum Sci. 2023 Aug 25;5:e26. doi: 10.1017/ehs.2023.21 (PMC10565192; doi:10.1017/ehs.2023.21)
Supplement: Supplementary file 1 [file ehssup.zip › S2513843X2300021Xsup001.zip!/images/220927_1_varEnv_trait_abundance_plot_all0_IP56AKN.pdf]

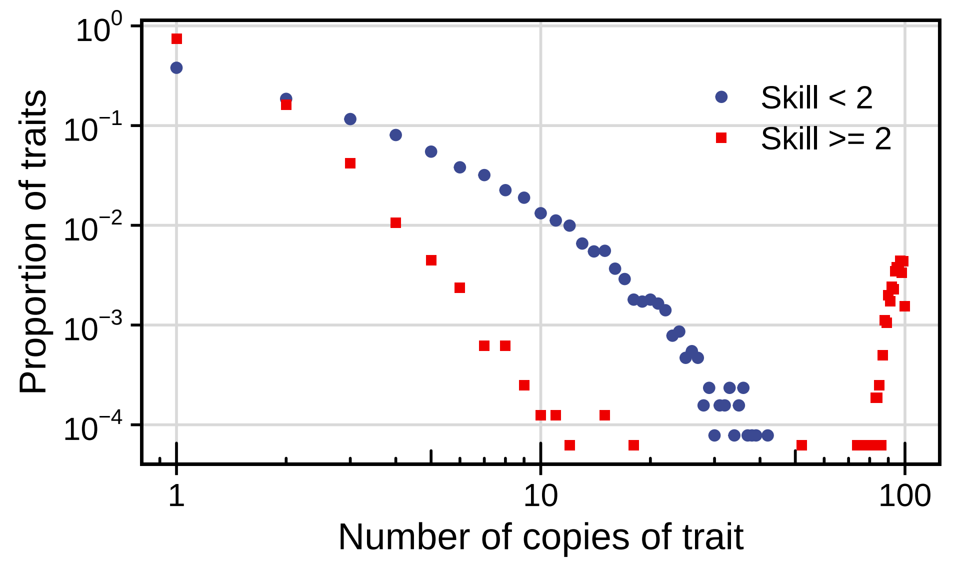

Proportion of traits
Number of copies of trait
Skill < 2
Skill >= 2
10^{0}
10^{-1}
10^{-2}
10^{-3}
10^{-4}
1
10
100
