# Supplementary material for: Pathways to cultural adaptation: the coevolution of cumulative culture and social networks
Source: Evol Hum Sci. 2023 Aug 25;5:e26. doi: 10.1017/ehs.2023.21 (PMC10565192; doi:10.1017/ehs.2023.21)
Supplement: Supplementary file 1 [file ehssup.zip › S2513843X2300021Xsup001.zip!/images/payoff_distribution_DA42ZYF.pdf]

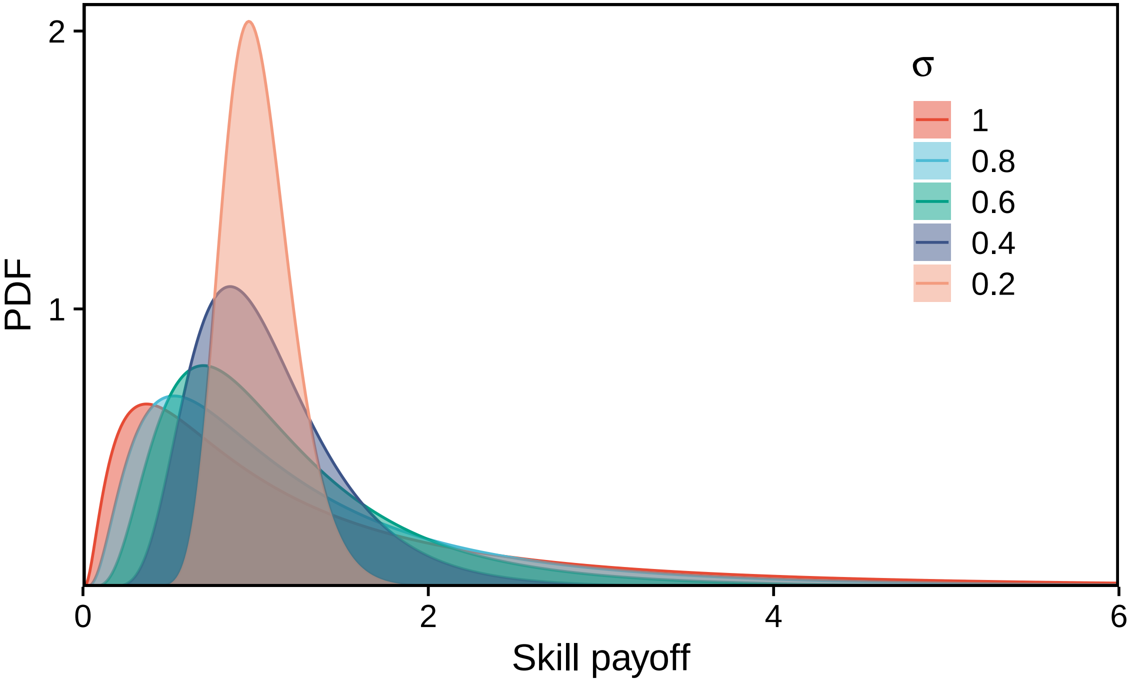

σ
1
0.8
0.6
0.4
0.2
PDF
2
1
0
2
4
6
Skill payoff
