# Supplementary material for: Pathways to cultural adaptation: the coevolution of cumulative culture and social networks
Source: Evol Hum Sci. 2023 Aug 25;5:e26. doi: 10.1017/ehs.2023.21 (PMC10565192; doi:10.1017/ehs.2023.21)
Supplement: Supplementary file 1 [file ehssup.zip › S2513843X2300021Xsup001.zip!/images/230616_1_changing_mutation_rate_scatter_all0_diff_mu_PO95UCJ.pdf]

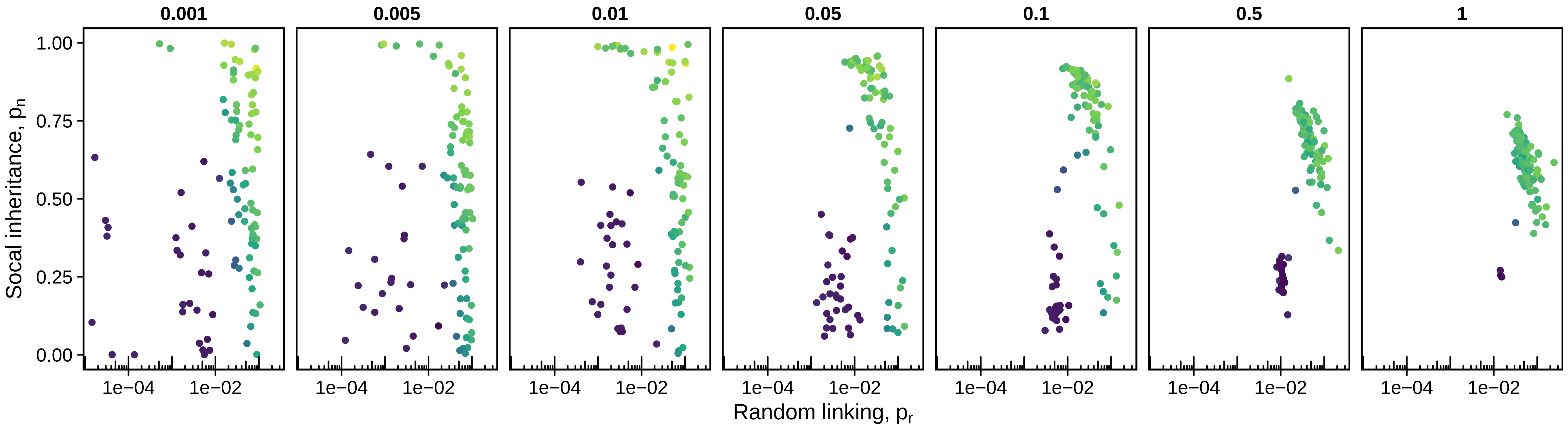
0.001
0.005
0.01
0.05
0.1
0.5
1
1.00
0.75
0.50
0.25
0.00
Socal inheritance, p_n
1e−04
1e−02
Random linking, p_r
