# Supplementary material for: Pathways to cultural adaptation: the coevolution of cumulative culture and social networks
Source: Evol Hum Sci. 2023 Aug 25;5:e26. doi: 10.1017/ehs.2023.21 (PMC10565192; doi:10.1017/ehs.2023.21)
Supplement: Supplementary file 1 [file ehssup.zip › S2513843X2300021Xsup001.zip!/images/230208_2_fertility_scatter_all0_ET82LDY.pdf]

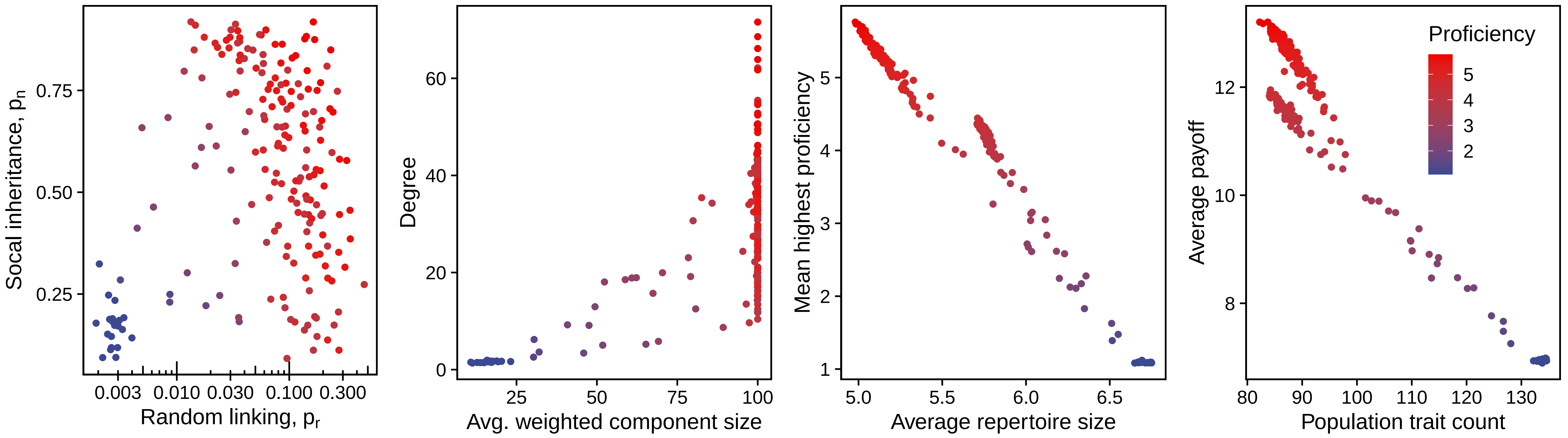

Socal inheritance, p_n
0.75
0.50
0.25
0.003
0.010
0.030
0.100
0.300
Random linking, p_r
Degree
60
40
20
0
25
50
75
100
Avg. weighted component size
Mean highest proficiency
5
4
3
2
1
5.0
5.5
6.0
6.5
Average repertoire size
Average payoff
12
10
8
80
90
100
110
120
130
Population trait count
Proficiency
5
4
3
2
