# Supplementary material for: Pathways to cultural adaptation: the coevolution of cumulative culture and social networks
Source: Evol Hum Sci. 2023 Aug 25;5:e26. doi: 10.1017/ehs.2023.21 (PMC10565192; doi:10.1017/ehs.2023.21)
Supplement: Supplementary file 1 [file ehssup.zip › S2513843X2300021Xsup001.zip!/images/220928_3_popSize_plot_lines_SE32JLP.pdf]

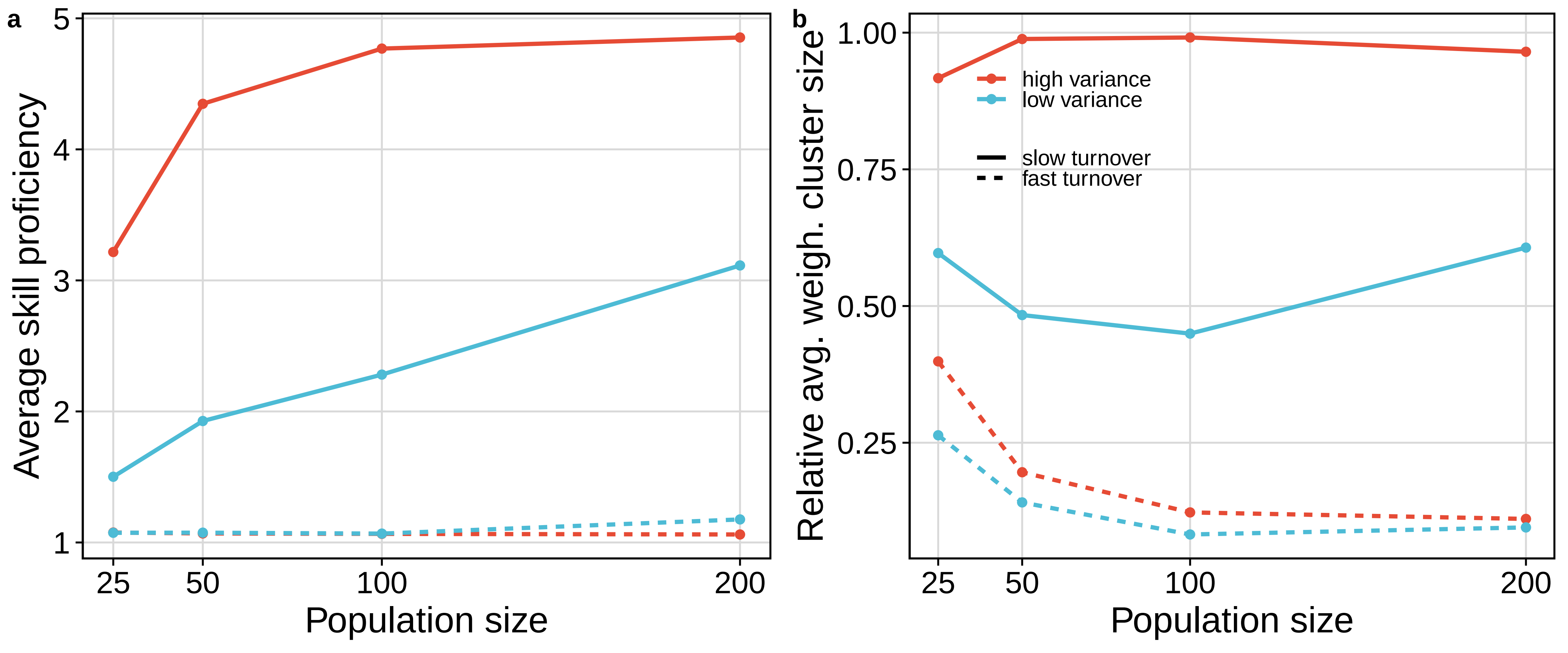
a
b
Average skill proficiency
Relative avg. weigh. cluster size
Population size
Population size
high variance
low variance
slow turnover
fast turnover
1
2
3
4
5
0.25
0.50
0.75
1.00
25
50
100
200
