# Supplementary material for: Pathways to cultural adaptation: the coevolution of cumulative culture and social networks
Source: Evol Hum Sci. 2023 Aug 25;5:e26. doi: 10.1017/ehs.2023.21 (PMC10565192; doi:10.1017/ehs.2023.21)
Supplement: Supplementary file 1 [file ehssup.zip › S2513843X2300021Xsup001.zip!/images/230112_1_pips_homo_ilsl_plots_XB27NUY.pdf]

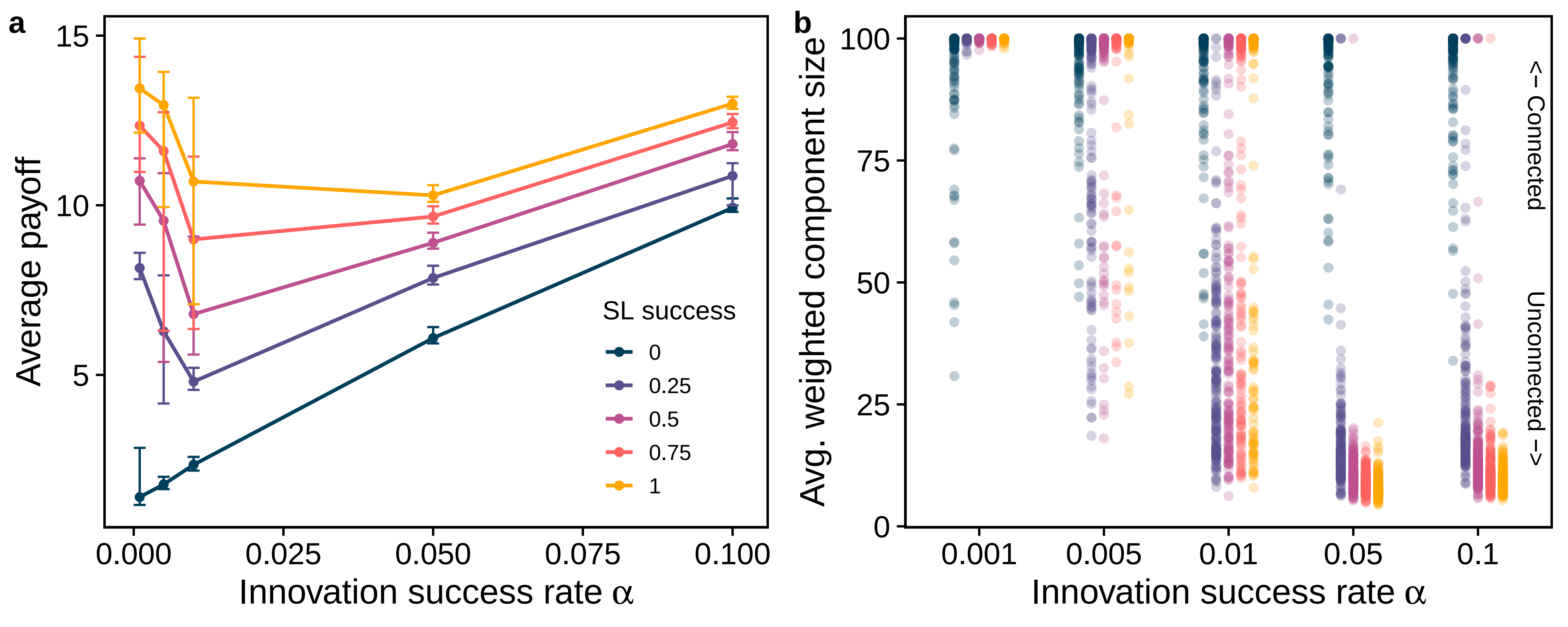

a
Average payoff
15
10
5
0.000
0.025
0.050
0.075
0.100
Innovation success rate α
SL success
0
0.25
0.5
0.75
1
b
Avg. weighted component size
100
75
50
25
0
0.001
0.005
0.01
0.05
0.1
Innovation success rate α
<-- Connected
Unconnected -->
