# Supplementary material for: Pathways to cultural adaptation: the coevolution of cumulative culture and social networks
Source: Evol Hum Sci. 2023 Aug 25;5:e26. doi: 10.1017/ehs.2023.21 (PMC10565192; doi:10.1017/ehs.2023.21)
Supplement: Supplementary file 1 [file ehssup.zip › S2513843X2300021Xsup001.zip!/images/230220_4_test_simpleContagion_env_false_colours_NK23YRZ.pdf]

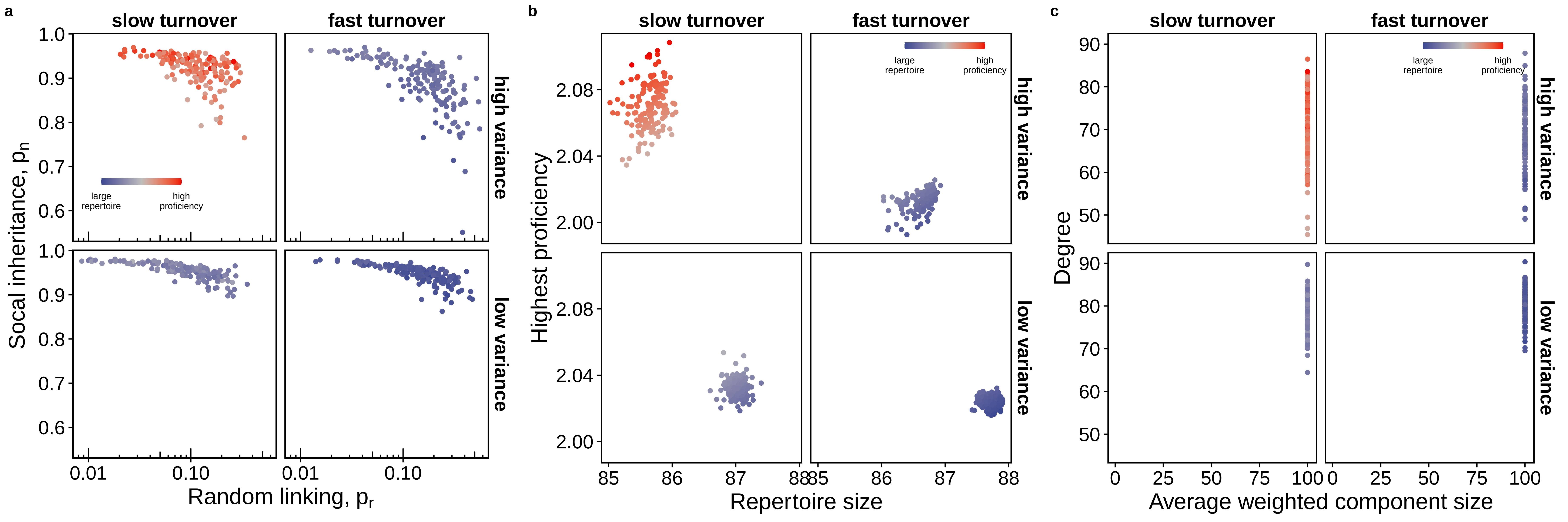

a
slow turnover
fast turnover
high variance
low variance
Socal inheritance, p_n
Random linking, p_r
large repertoire
high proficiency
b
slow turnover
fast turnover
high variance
low variance
Highest proficiency
Repertoire size
large repertoire
high proficiency
c
slow turnover
fast turnover
high variance
low variance
Degree
Average weighted component size
large repertoire
high proficiency
